# Cannabinoid Actions on Neural Stem Cells: Implications for Pathophysiology

**DOI:** 10.3390/molecules24071350

**Published:** 2019-04-05

**Authors:** Rui S. Rodrigues, Diogo M. Lourenço, Sara L. Paulo, Joana M. Mateus, Miguel F. Ferreira, Francisco M. Mouro, João B. Moreira, Filipa F. Ribeiro, Ana M. Sebastião, Sara Xapelli

**Affiliations:** 1 Instituto de Farmacologia e Neurociências, Faculdade de Medicina, Universidade de Lisboa, Lisboa, 1649-028 Lisboa, Portugal; rmsrodrigues@medicina.ulisboa.pt (R.S.R.); diogo.lourenco@medicina.ulisboa.pt (D.M.L.); sara.paulo@medicina.ulisboa.pt (S.L.P.); joana.mateus@medicina.ulisboa.pt (J.M.M.); jorge.ferreira@medicina.ulisboa.pt (M.F.F.); fmouro@medicina.ulisboa.pt (F.M.M.); joaomoreira@medicina.ulisboa.pt (J.B.M.); anaribeiro@medicina.ulisboa.pt (F.F.R.); anaseb@medicina.ulisboa.pt (A.M.S.); 2 Instituto de Medicina Molecular João Lobo Antunes, Faculdade de Medicina, Universidade de Lisboa, Lisboa, 1649-028 Lisboa, Portugal

**Keywords:** cannabinoids, neural stem cells, neurogenesis, regeneration, pathophysiology

## Abstract

With the increase of life expectancy, neurodegenerative disorders are becoming not only a health but also a social burden worldwide. However, due to the multitude of pathophysiological disease states, current treatments fail to meet the desired outcomes. Therefore, there is a need for new therapeutic strategies focusing on more integrated, personalized and effective approaches. The prospect of using neural stem cells (NSC) as regenerative therapies is very promising, however several issues still need to be addressed. In particular, the potential actions of pharmacological agents used to modulate NSC activity are highly relevant. With the ongoing discussion of cannabinoid usage for medical purposes and reports drawing attention to the effects of cannabinoids on NSC regulation, there is an enormous, and yet, uncovered potential for cannabinoids as treatment options for several neurological disorders, specifically when combined with stem cell therapy. In this manuscript, we review in detail how cannabinoids act as potent regulators of NSC biology and their potential to modulate several neurogenic features in the context of pathophysiology.

## 1. Introduction

The development of a multicellular organism can be compared to a choreographed dance of cellular and molecular interactions involving cell reorganization during precise stages. Although in most regions of the mammalian brain the production of neurons is largely confined to the prenatal period, in specific brain regions, neurogenesis occurs postnatally and continues into adulthood.

In the mammalian central nervous system (CNS), neural stem cells (NSCs) are characterized by their self-renewal capability and multipotency, i.e., the ability to give rise to both neurons and glial cells, such as oligodendrocytes and astrocytes. A ‘‘neurogenic niche’’ can be defined as a complex microenvironment that supports NSCs and their progeny, helping to determine whether NSCs remain dormant or divide, by providing signals that guide early stages of proliferation or differentiation. One of these signals has been shown to come through the action of endocannabinoids (eCBs), mainly via activation of cannabinoid receptors type 1 and 2 (CB1R and CB2R). Cannabinoid research has been capturing the interest of physicians, researchers, pharmaceutical companies and of the general population worldwide because of its broad range of applications. Importantly, increasing data has been showing an important role for cannabinoids in NSC modulation, which might allow combining their wide range of actions with the multitude of applications that stem cells offer. 

In this review, we provide a summary of cannabinoid actions and its effects in NSC modulation both in development and in the adult brain, highlighting the role of cannabinoids in pathophysiology and as therapeutic agents for neuroregeneration.

## 2. Endocannabinoid System and Cannabinoids

Cannabis has long been used by humans due to its therapeutic value, for recreational and religious purposes, to produce food for livestock and, for its fibers, to manufacture clothing [1]. Nowadays, a growing body of scientific evidence has been attesting the immense potential of this plant to ameliorate symptoms of several diseases. Indeed, medical-cannabis is being used or proposed to treat neuropathic pain and muscle spasticity associated with multiple sclerosis (MS), neurodevelopmental forms of refractory epilepsy, neurodegenerative and chronic diseases [1,2,3,4,5,6,7,8,9,10]. Additionally, there is strong scientific support for its use in eating disorders, to reduce vomiting and nausea associated with chemotherapy, and to alleviate human immunodeficiency virus infection and acquired immune deficiency syndrome (HIV/AIDS) related weight loss [1]. 

On the other hand, chronic consumption or therapeutic exposure to cannabis can be related with detrimental health effects. Specifically, heavy and sustained cannabis use is associated with cognitive and memory impairments, increased probability of developing schizophrenia-spectrum disorders, acute psychosis and mania [11,12,13,14,15]. Regular cannabis abuse can result in chronic bronchitis and impaired respiratory function if consumed through inhalation. It can also induce physical and significant mental dependence, tolerance and withdrawal symptoms [1,16]. Therefore, one of the challenges of cannabis research is to find ways to prevent the negative side-effects associated with cannabis-based medicines [17,18].

According to the World Drug Report 2017, marijuana (dried leaves, flowers, stems and seeds from the *Cannabis sativa* or *Cannabis indica* plants) is consumed by up to 238 million people worldwide, making it, by far, the most widely used drug [19]. The psychoactive effects of cannabis consumption include euphoria, appetite stimulation, sedation, altered perception, impairments in motor control and memory deficits [20]. These effects are almost exclusively related with the presence of Δ^9^-tetrahydrocannabinol (Δ^9^-THC), which was firstly isolated in its pure form and structurally described in 1964 [21]. Regardless of its psychoactive effects, Δ^9^-THC has therapeutic value and unique applications [22].

More than 120 phytocannabinoids (natural occurring cannabinoids) have now been identified as constituents of the cannabis plant [23]. Besides Δ^9^-THC, the most abundant cannabinoids present in the cannabis plant are Δ^8^-tetrahydrocannabinol (Δ^8^-THC), cannabinol (CBN), cannabidiol (CBD), cannabigerol (CBG), cannabichromene (CBC), Δ^9^-tetrahydrocannabivarin (THCV), cannabivarin (CBV) and cannabidivarin (CBDV) [23].

### 2.1. Endocannabinoid System

The endocannabinoid system (ECS) is a phylogenetically old modulatory system, found in both vertebrate and invertebrate species [24,25,26]. The ECS encompasses eCB molecules, amongst which the two best known and characterized are *N*-arachidonoylethanolamine (anandamide, AEA) and 2-arachidonoglycerol (2-AG), their synthetizing and degrading enzymes, the two major cannabinoid receptors CB1R and CB2R, the endocannabinoid membrane transporter (EMT) and the CB1R interacting protein 1a (CRIP1a) [1]. 

**Figure 1:**
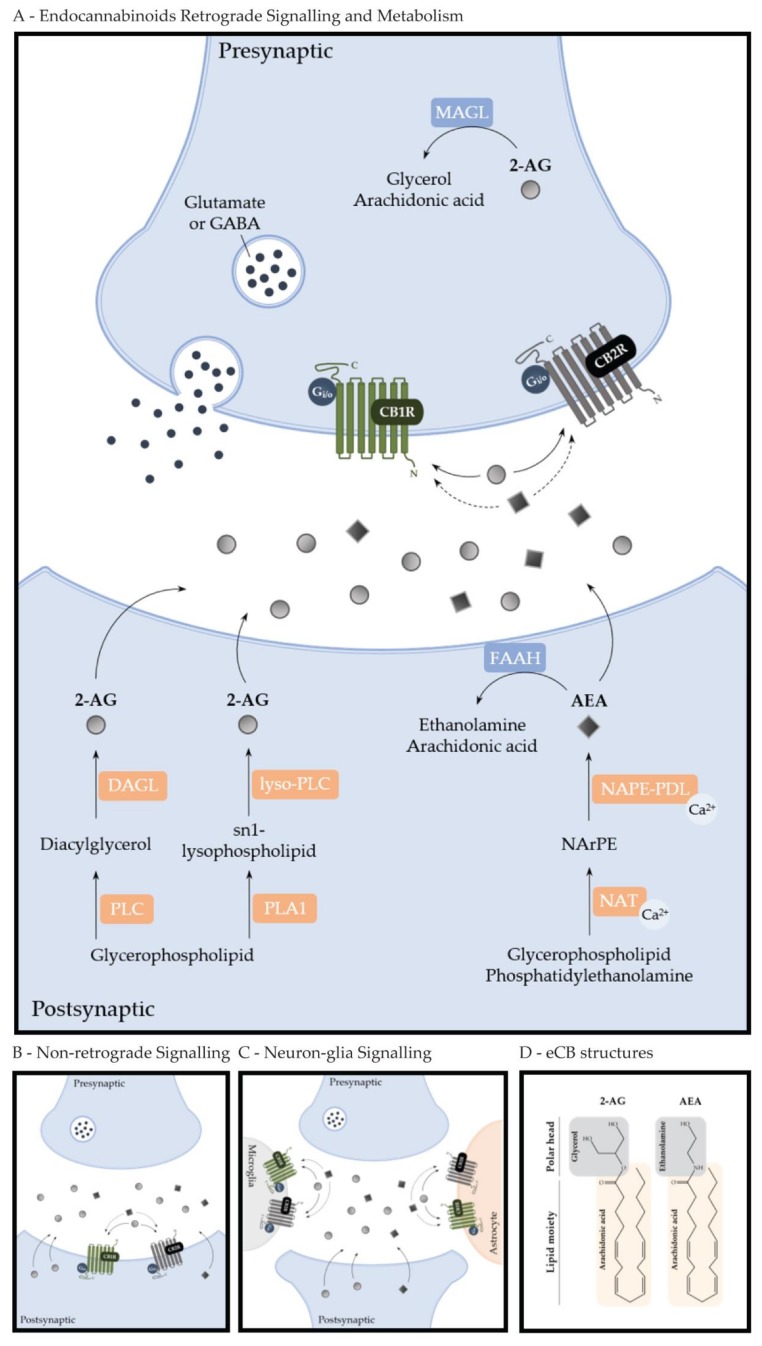
Endocannabinoid signaling. (**A**) Endocannabinoid Retrograde Signaling and Metabolism. 2-arachidonoglycerol (2-AG) is synthesized by two different metabolic pathways: via the cleavage of diacylglycerol by diacylglycerol lipase (DAGL), where diacylglycerol is released from membrane phospholipids by phospholipase C (PLC) or via the action of phospholipase A1 (PLA1), releasing an sn-1 lysophospholipid from membrane phospholipids, which is cleaved by lyso-PLC in order to generate 2-AG. On the other hand, a calcium-dependent trans-acylase (NAT) acts on glycerophospholipids and phosphatidylethanolamine, resulting in *N*-arachidonoyl-phosphatidyl ethanolamine (NArPE), which is then cleaved by a calcium-dependent NAPE (*N*-acyl-phosphatidylethanolamine)-specific phospholipase D (NAPE-PLD), releasing *N*-arachidonoylethanolamine (anandamide, AEA) from membrane lipids. While hydrolysis of AEA occurs postsynaptically, via fatty acid amide hydrolase (FAAH) into arachidonic acid and ethanolamine, 2-AG is hydrolyzed by monoacylglycerol lipase (MAGL) into arachidonic acid and glycerol presynaptically. AEA and 2-AG are usually synthesized postsynaptically and are released “on demand” to the synaptic cleft, where they modulate presynaptic glutamatergic or GABAergic signaling by binding to CB1R or CB2R. (**B**) Non-retrograde Signaling. AEA and 2-AG signal autocrinally and non-retrogradely, the postsynaptic neuron, modulating synaptic transmission. (**C**) Neuron-glia Signaling. Endocannabinoids produced by neurons can bind to the cannabinoid receptors expressed in astrocytes and microglia. This neuron-glia signaling is able to modulate several responses. (**D**) eCB structures. 2-AG and AEA have similar molecular structures. They are both polar ester lipids formed by the bond of the omega-6 fatty acid arachidonic acid with either glycerol (to form 2-AG) or ethanolamine (to form AEA).

The first identified eCB was AEA [1]. Briefly after, two other eCBs were identified, namely 2-AG and 2-arachidonoyl glyceryl ether, commonly known as noladin. eCBs are equivalent regarding the presence of a polyunsaturated fatty acid moiety (such as the arachidonic acid) and a polar head group, composed by ethanolamine or glycerol (Figure 1) [27,28,29,30]. 

AEA is known to act as a partial agonist of CB1Rs and CB2Rs, while 2-AG as a full agonist of both receptors. AEA is synthesized via the action of a calcium-dependent trans-acylase (NAT) on phosphoglycerides and phosphatidylethanolamine. The resulting *N*-arachidonoyl-phosphatidyl ethanolamine (NArPE) is then cleaved by a calcium-dependent NAPE (*N*-acyl-phosphatidylethanolamine)-specific phospholipase D (NAPE-PLD), releasing the AEA from membrane lipids. This eCB is hydrolyzed into arachidonic acid and ethanolamine by fatty acid amide hydrolase (FAAH) [31]. 2-AG, on the other hand, can be synthesized by two different metabolic pathways: via the cleavage of diacylglycerol by diacylglycerol lipase (DAGL), where diacylglycerol is released from membrane phospholipids by phospholipase C (PLC) or via the action of phospholipase A1 (PLA1), releasing an sn-1 lysophospholipid from membrane phospholipids, which is cleaved by lyso-PLC in order to generate 2-AG. This eCB is hydrolyzed by monoacylglycerol lipase (MAGL) into arachidonic acid and glycerol (Figure 1A) [31]. These molecules are not stored in vesicles but rather are synthetized and released “on demand,” with AEA being less abundant than 2-AG [1,32].

eCBs display a broad spectrum of physiologic relevant roles, particularly in the CNS and peripheral nervous system (PNS) [30,33]. These roles are mainly mediated through the activation of CB1Rs and CB2Rs, both being G protein-coupled seven transmembrane domain receptors (GPCR) [33]. A common feature usually associated with CB1R and CB2R activation is the modulation of either spontaneous or evoked release of chemical messengers, although this effect is much better characterized for CB1Rs [34]. eCBs are produced and released from postsynaptic neurons either phasically (in an activity-dependent manner), or tonically (under basal conditions). The released eCBs then act retrogradely by activating presynaptic receptors (Figure 1A) [33,35]. Hence, regulation of neurotransmitter release constitutes a major physiological role of the ECS [30]. Indeed, eCBs are involved in important forms of short and long-term plasticity, by suppressing neurotransmitter release transiently (short-term depression, STD) or persistently (long-term depression, LTD), mainly through the activation of presynaptic CB1Rs [33]. Importantly, eCBs can control both inhibitory synaptic transmission (a process designated as depolarization-induced suppression of inhibition, DSI) and excitatory synaptic transmission (the depolarization-induced suppression of excitation, DSE) [36,37]. 

Available evidence also suggests that eCB signaling can occur in a non-retrograde mode, through autocrine signaling. Specifically, eCBs can modulate synaptic transmission through direct activation of transient receptor potential vanilloid receptor type 1 (TRPV1R), in which AEA is known to act as a full agonist, or via postsynaptically located CB1Rs (Figure 1B) [38,39].

The abundancy of CB1Rs in the brain strongly supports that this receptor is responsible for the majority of the psychoactive effects of exogenous cannabinoids and for the physiological actions of eCBs [25,33]. This is further supported by the fact that selective CB1R antagonists effectively abolish the psychoactive effects of these drugs [40]. As reviewed by Solymosi and Köfalvi (2017), the CB1R is abundantly present in the CNS, with higher densities found in the substantia nigra pars reticulata, globus pallidus, entopeduncular nucleus, inner granule cell layer (GCL) of the olfactory bulb, layers I-III, Va and VI of the cerebral cortex, hippocampus (particularly in the molecular layer and Cornu Ammonis 3 (CA3) region) and dorsolateral striatum; moderate levels of CB1Rs can be found in the hypothalamus, ventral striatum/nucleus accumbens, septum and amygdala [1,41,42].

CB1R activation has been linked to neuroprotection by controlling excessive excitatory transmission and calcium release, thus protecting synapses from excitotoxity [43]. Although being classically viewed as a G_i/o_-coupled receptor, hence having mainly inhibitory actions, CB1R coupling to G proteins is mutable [30,33,44]. Indeed, CB1R is now believed to have few “intrinsic” signaling properties, and the actions mediated by its activation are largely dependent on cell type, location, functional state and temporal constrains (for a detailed review see [44]). All these nuances can drastically modify the effects of endo-, exo- and synthetic cannabinoids. 

Contrary to initial belief that CB2Rs were exclusively present in the periphery, particularly in the immune system, such as in the spleen, leukocytes and tonsils, these receptors were later identified in microglial cells [33,45,46,47,48]. New and more advanced technological approaches have allowed the identification of CB2Rs in astrocytes and, to a larger degree, in neurons (Figure 1C) [49,50,51]. In the CNS, they were first identified in brainstem neurons and later in inhibitory and excitatory neurons in the hippocampus [52,53]. Nowadays, CB2Rs are known to be expressed in the presynaptic terminals of gaminobutyric acid-containing (GABAergic) interneurons in the hippocampus and medial entorhinal cortex, and in cortical and hippocampal astrocytes, where CB2R activation leads to higher glucose uptake [54,55]. CB2R levels can be increased in neurons and astrocytes following specific insults (such as neuroinflammation) and also in certain disease states [56,57,58]. Additionally, considering the lack of psychoactive effects following CB2R modulation, this receptor is becoming popular as a very promising therapeutic target [46]. 

When taking into account the psychoactive and therapeutic properties of cannabinoids, it is important to consider that the phytocannabinoids existent in the cannabis plant, and in other plants, affect brain activity by coupling to other receptors beyond the classic CB1Rs and CB2Rs [1,23]. Being so, serotonin 5HT1-3A receptors (5HT1AR, 5HT2AR, 5HT3AR), G protein-coupled receptors 18 and 55 (GPR18, GPR55), transient receptor potential (TRP) family, glycine receptors and the peroxisome proliferator-activated receptor (PPAR) assume a particular importance when considering the possible therapeutic applications of cannabinoids [1,23]. Anatomical and functional co-localization studies have shown that these receptors are expressed in the same regions as CB1Rs and CB2Rs in the CNS and PNS [59,60,61,62].

### 2.2. Cannabinoid Pharmacology and Actions

Δ^9^-THC and CBD are the two best known and characterized phytocannabinoids. The cataleptic effect of Δ^9^-THC was the first psychoactive effect described of a cannabinoid [63]. This effect is used as a standard of cannabinoid psychoactivity, with which it has been shown to be strongly correlated [64].

Δ^9^-THC is known to act as a partial agonist of CB1Rs and CB2Rs, presenting a mixed agonist-antagonist profile depending on the cell type, concentration, receptor expression and presence of other endo- and exo-cannabinoids acting as full agonists [23,65,66]. As reviewed by Solymosi and Köfalvi (2017), Δ^9^-THC can have different coupling profiles [1]. In fact, some reports show that Δ^9^-THC can be more potent on the CB2Rs and, paradoxically, at higher concentrations it may even act as an antagonist of CB1Rs [65,67,68]. Δ^9^-THC is considered a “classic cannabinoid” because it passes the mouse tetrad bioassay through the activation of CB1Rs, by eliciting the cannabinoid-induced tetrad (hypothermia, hypolocomotion, catalepsy and analgesia) [64,69]. As thoroughly reviewed by Solymosi and Köfalvi (2017), besides its actions on CB1Rs and CB2Rs, Δ^9^-THC binds to several other receptors such as GPR55, as well as serotonin, opioid, glycine and PPARγ receptors, which may account for some of the effects described for this phytocannabinoid [1,23]. However, Δ^9^-THC has been shown to have no effect on the TRPV1R, although it acts on other TRPV channels (for detailed reviews see [1,23]).

CBD is a non-psychoactive cannabinoid, therefore, has high medicinal value, being widely studied. This cannabinoid has been shown to act as an anti-inflammatory, antioxidant, anti-epileptic, antirheumatic, anxiolytic and analgesic drug [1,23]. CDB was also shown to reduce congestion and nausea and to be neuroprotective [1,23]. Furthermore, CBD positively modifies the effects of Δ^9^-THC by reducing its psychoactive effects and by increasing its clinical efficacy and the duration of its beneficial effects [1,22]. CBD was early characterized as absent of cataleptic effects, which prompted the now well accepted evidence that CBD is devoid of psychoactive effects [63,64]. 

As reviewed by Solymosi and Köfalvi (2017), CBD has low affinity to CB1Rs and CB2Rs, exhibiting no agonist activity [1,67,70]. On the contrary, in vitro studies have shown that CBD has surprisingly high potency as an antagonist of both CB1Rs and CB2Rs [71]. Recently, however, it is being proposed that CBD may actually act as a negative allosteric modulator of CB1Rs [72]. Furthermore, it inhibits the cellular reuptake of AEA, directly affecting eCB tone [23]. It also acts as an agonist of GPR55 and as an antagonist of GPR18 [68,73,74]. In a recent work, the anti-inflammatory and immunosuppressive effects of CBD were proposed to be linked with its ability to activate adenosine type 1 receptors [75]. CBD effects on TRPV, glycine, GABA_A_ and PPARγ receptors were described in detail in [23]. 

eCBs, phytocannabinoids such as Δ^9^-THC and CBD, as well as other synthetic cannabinoids, were shown to modulate embryonic and postnatal neurogenesis. In the next sections of this review, we will further explore the role of the ECS in NSC modulation and its implications for pathophysiology. 

## 3. Neurogenesis

### 3.1. Neurodevelopmental Neurogenesis

During embryonic development, neurogenesis can be distinguished in two different stages that, although having different cellular and molecular mechanisms, occur simultaneously. These are neurulation and embryonic neurogenesis.

#### 3.1.1. Neurulation

**Figure 2:**
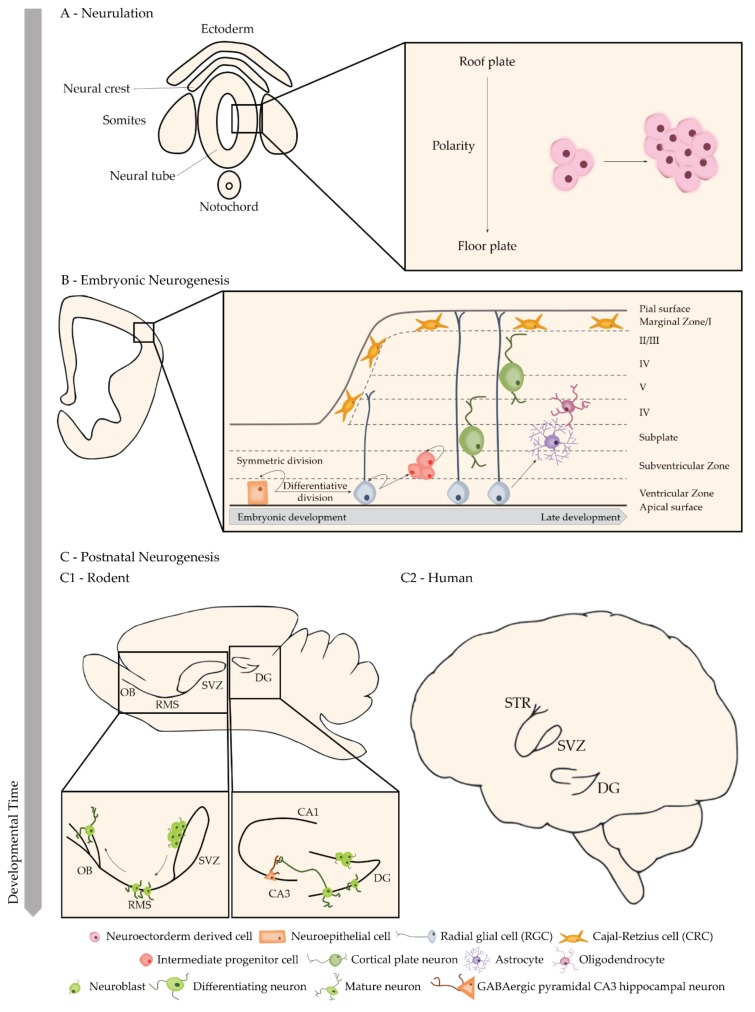
Neurogenesis. (**A**) Neurulation. Schematic representation of the neurulation process. Neuroectoderm derived cells proliferate at different rates along the apicobasal polarity. (**B**) Embryonic Neurogenesis. During embryonic development the central nervous system develops in an inside-out fashion. Neuroepithelial stem cells, via asymmetrical divisions, differentiate into radial glial cells (RGCs), establishing the Ventricular Zone near the apical surface. Near the pial surface, Cajal- Retzius (CRCs) cells will make the marginal zone during embryogenesis and the cortical layer I postnatally. The other cortical layers are formed in sequential waves during neurogenesis. Later in development, neural progenitors enter a gliogenic mode, generating astrocytes and oligodendrocytes. Neuroepithelial, radial glial and intermediate progenitor cells are capable of symmetric and asymmetric divisions. (**C**) Postnatal Neurogenesis. In the adult brain, neural stem cells can be found in two neurogenic niches, the Subventricular Zone (SVZ) and in the Subgranular cell layer of the Dentate Gyrus (DG). In rodents (**C1**), after differentiating in the SVZ, immature neurons migrate along the rostral migratory stream (RMS) to the olfactory bulb (OB) where they will mature mainly into GABAergic interneurons. On the other hand, in humans (**C2**), immature neurons are thought to migrate along the RMS to the striatum (STR). In rodents and humans, differentiating neurons from the DG mature by making functional connections with pyramidal CA3 hippocampal neurons.

Arising from the ectoderm, one of the three primary germ layers, the CNS begins developing when the neural plate folds into the neural tube, through a process called neurulation, giving origin to the brain in the rostral region and the spinal cord in the caudal region [76]. During this stage there is an increase in the number of neuroectoderm-derived proliferating cells, expanding the neural epithelium at different rates in order to form more specialized regions of the mature CNS [77]. These then become the forebrain, the midbrain and the hindbrain. Simultaneously, several factors are released from the notochord and the somites around the neural tube, establishing a dorsoventral polarity [78]. This polarity is further reinforced by the formation of the anterior-posterior axis through the Wnt signalling gradient (Figure 2A) [79].

#### 3.1.2. Embryonic Neurogenesis

Neuroepithelial progenitor cells (NECs) can be found lining inside the neural tube, which will develop into the ventricles [80]. They form a pseudostratified epithelium, as their nuclei migrate with the cell-cycle stage (interkinetic nuclear migration), being near the apical side during mitosis and more basally during the S phase [81]. Like other stem cells, NECs divide in either a symmetric proliferative manner, in which both daughter cells remain mitotic, or in a differentiative manner, in which at least one daughter cell exits the cell cycle and differentiates into a more specialized cell [82]. This foundational process forms several layers surrounding the lumen of the developing nervous system, the inner-most apical layer, where the progenitor cells reside, being the ventricular zone (VZ). Radial glial cells (RGCs) are the earliest type of cells to be distinguishable within the neural epithelium. The cell bodies of these cells can be found in the VZ and their long processes extend outwards, to the pial surface [83]. Outer RGCs and intermediate progenitors will then establish the subventricular zone (SVZ), which becomes one of the adult neurogenic niches. RGCs will originate transit amplifying cells to increase neuronal production [83,84]. NECs exit the cell cycle near the ventricular surface and invade the preplate (PP—a primordial plate above the VZ). Migrating neurons move past the subplate, displacing this layer away from the Cajal-Retzius cells (CRCs), which remain adjacent to the pial surface of the developing brain in a cell-sparse area known as the marginal zone [85]. Distinct projection neuron subtypes are born in sequential waves over the course of neurogenesis [86]. As new cortical plate neurons arrive to the RGCs, they migrate past the older subplate and cortical plate neurons before inserting beneath CRCs [87]. This inside-out arrangement of projection neurons makes the oldest neurons (cortical layer VI) closest and the youngest neurons (cortical layer II/III) farthest from their birthplace near the ventricle (Figure 2B). This cortical expansion, despite being conserved within all mammalian species, is different whether organisms have a smooth (lissencephalic, e.g., mouse, rat) or highly convoluted (gyrencephalic, e.g., ferret, human) neocortex [88,89]. One key difference is associated with the size of the neocortex, which can be better correlated with its surface area than with its thickness, which only varies slightly across species [90]. Brain convolutions appeared as an evolutionary solution to the problem of increasing cortical surface area (e.g., neuron numbers) without prohibitively increasing the size of the skull [91]. Another striking interspecies difference is related to the expansion of the basal progenitors [92]. In gyrencephalic species, the SVZ can be further dissected in two distinct germinal zones: the inner SVZ, which largely resembles the SVZ of lissencephalic rodents, and the outer SVZ, which is absent in most lissencephalic species [92]. This subdivision of the SVZ has a tremendous impact on the number of basal progenitors contained in each layer. Indeed, during peak stages of embryonic neurogenesis, the outer SVZ harbors up to four times as many progenitors as the VZ and inner SVZ combined [93]. However, this does not correlate with a higher number of neurons in the adult brain. In fact, in later developmental stages, after embryonic neurogenesis is complete, neural progenitors transition into a gliogenic mode, generating astrocytes and oligodendrocytes [94,95]. CRCs will migrate into the neocortical layer I of the cortex from non-cortical locations, whereas projection neurons born in the neocortical VZ and SVZ, migrate along radial processes to reach their final laminar destinations in the cortex [96]. The presence of NSCs in most CNS regions decreases dramatically after embryonic development but, in many species, remains throughout life in localized neurogenic niches [97,98,99,100].

### 3.2. Adult Neurogenesis

Adult NSCs can be mostly found in two neurogenic niches, the SVZ and the subgranular zone (SGZ) of the hippocampal dentate gyrus (DG) (Figure 2C) [101]. The existence of self-renewing adult NSCs in the brain led to the hypothesis that NSCs are tri-potent, having the capacity to generate neurons, astrocytes, and oligodendrocytes. However, the pioneering in vivo studies in the adult hippocampus by Eriksson and colleagues have only found the generation of neurons and astrocytes, but not oligodendrocytes [102]. On the other hand, in vivo studies focusing on the adult SVZ suggest that NSCs can give rise to the three neural cell types, but whether neurons and glia arise from distinct stem cell lineages in vivo is still unknown [103]. 

Adult NSCs are mostly quiescent in vivo due to the specific cellular and molecular characteristics of the niches where they reside, allowing them to withstand metabolic stress and to preserve genome integrity over long periods of time [104,105]. The balance between the cytoarchitecture of the niches and the factors that regulate quiescence and activation of NSCs is so delicate that it has been proposed that tumorigenic or brain tumor stem cells may arise from an imbalance of those factors [106]. In fact, both NSCs and tumorigenic cells share many of the molecular pathways that regulate proliferation, such as sonic hedgehog (shh) or Wnt-signalling [106]. 

Despite having been shown to exist in several mammals since the 1960s (e.g., mice, rats, cats, song birds, treeshrews, marmosets, macaques), in humans, adult neurogenesis has sparked an intense debate regarding its existence [107,108,109,110,111,112]. Reports of human neurogenesis are mostly based on the analysis of neurogenic markers, namely doublecortin (DCX) and polysialic acid neural cell adhesion molecule (PSA-NCAM) on post-mortem brain samples [102,113,114,115]. It has been suggested that the differences found in studies where human adult neurogenesis is either proved or disproved could be due to several factors around the time of death, namely stress hormones, protein integrity and the general health state of subjects; or even the post-mortem delay between sample collection and preparation [116,117]. In fact, it has been shown in animal studies that the DCX signal becomes weak within a few hours of post-mortem delay, therefore time is a crucial factor on neurogenesis studies, impacting the overall analysis of data [118]. A recent study by Sorrells and colleagues (2018) showed that adult neurogenesis drops to undetectable levels with aging. This study was conducted with samples from patients with epilepsy, where normal hippocampal circuitry and neurogenesis is known to be disrupted and had a post-mortem delay of around 48 h between collection and fixation [113]. On the other hand, in Boldrini et al. (2018) and more recently in Moreno-Jiménez et al. (2019) studies, reported lifelong neurogenesis in humans. In these studies the post-mortem delay was no longer than 26 h and samples were collected from both control and pathological subjects [114,115]. Surprisingly, in Moreno-Jiménez et al. (2019) study, samples from patients with Alzheimer’s disease (AD) also have immature progenitor cells, i.e., DCX positive cells, although the number and maturation of these cells progressively declined as AD advanced, again reinforcing the idea that tissue handling is essential for the preservation and detection of neurogenic markers [115].

#### 3.2.1. Subventricular Zone

**Figure 3:**
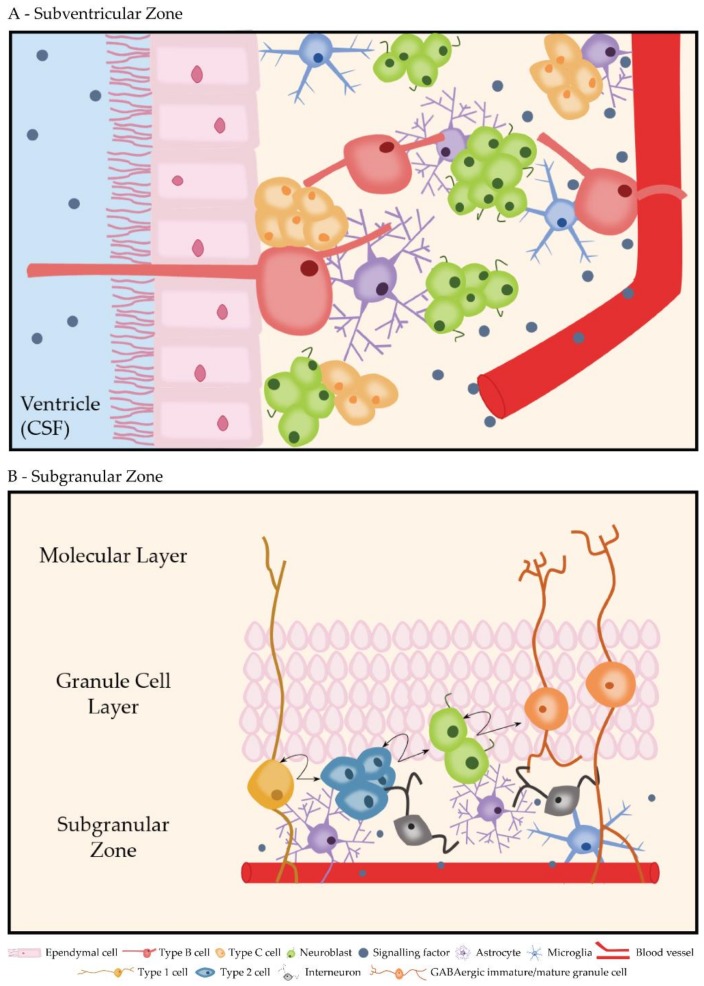
The neurogenic niches. (**A**) Cytoarchitecture of the Subventricular Zone. Type B cells extend cellular processes to the ventricle where they come into contact with cerebralspinal fluid (CSF) and to nearby blood vessels, detecting intrinsic and extrinsic factors that will signal for proliferation and/or differentiation. B cells differentiate into C cells, which generate neuroblasts. Astrocytes and microglia are also found in the neurogenic niche and support the stem cell pool and the development of newborn cells. (**B**) Cytoarchitecture of the Subgranular Zone. The dentate gyrus (DG) is composed of three layers: the molecular layer; the granule cell layer, which are densely packed granule cells; and the subgranular zone, containing neural stem and progenitor cells. Here, type 1 cells extend cellular processes to the molecular layer and can generate intermediate progenitor cells, or type 2 cells, which are closely associated with the hippocampal vasculature and the detection of signaling factors. Neuroblasts differentiate from type 2 cells and into GABAergic dentate granule cells. These mature by making functional connections with CA3 hippocampal cells, whilst migrating towards the molecular layer of the DG. Interneurons, microglia and astrocytes contribute to the maintenance and support of the niche.

Ependymal cells are organized into rosette shaped structures bordering the SVZ which is lining the lateral wall of the lateral ventricles. There are three cell types that mainly compose the SVZ niche: B cells, C cells and A cells. B cells (or radial glia-like NSCs) extend radial processes to contact with blood vessels and a single cilium through the ependymal rosettes to contact the cerebrospinal fluid in the ventricular space [119]. These processes allow the detection of both intrinsic (e.g., shh, Wnt or Notch-signaling) and extrinsic factors (e.g., neurotransmitters, hormones or growth factors) that will signal for either proliferation, differentiation, or both [120]. B cells can divide and differentiate into C cells (or transit amplifying cells), which then generate A cells (or neuroblasts) [121]. In rodents, neuroblasts migrate down the rostral migratory stream (RMS) to the olfactory bulb where they differentiate mainly into GABAergic interneurons and are integrated either in the GCL or in the periglomerular layer. Thus, SVZ neurogenesis plays an important role in the neuroplasticity and olfactory memory [122,123]. Importantly, in the case of brain injury, neuroblasts can also migrate to lesion sites where they differentiate into other types of neural cells [124,125]. Additionally, astrocytes and microglia are also present in the SVZ contributing to the cellular architecture of the niche (Figure 3A) [104]. 

Several reports have shown that in humans, neurogenesis in the SVZ is slightly different when compared with rodent models [126,127]. One key aspect regarding these differences is the RMS, which is shorter in humans due to the extensive frontal lobe development [128]. These anatomical characteristics are also consistent with observations in other primate brains [129]. Another difference described is directly related with the way neuroblasts migrate along the RMS. It has been suggested that glial tubes can support the proliferation and migration of neuroblasts. In rodents, glial fibrillary acidic protein (GFAP) positive glial cells form tubes and, within these glial tubes, neuroblasts pack in chain-like structures [130]. In humans, however, there is a ‘meshwork’ of glial cells supporting the migration of neuroblasts, most likely to the striatum [131,132].

#### 3.2.2. Subgranular Zone

The hippocampal DG can be dissected into two layers, the GCL and the molecular layer (ML) [133]. The SGZ is found underneath the GCL, where radial glia-like NSCs (or type 1 cells) reside and extend processes from the GCL to the ML [134]. These cells generate intermediate progenitor cells (IPCs, or type 2 cells), which generate neuroblasts (or type 3 cells) that differentiate into mostly GABAergic mature granule cells (GCs), which then migrate into the GCL of the DG [135]. Type 2 cells are closely associated with the vasculature of the hippocampus [136]. In addition to the aforementioned cell types, astrocytes, microglia, and interneurons contribute to the cellular architecture of the niche (Figure 3B) [137]. It is theorized that the rapid maturation of newly formed GCs is responsible for the creation of new memory representations and their storage in the circuit [133]. This process is possible because of the high levels of excitability of new GCs which, in early stages of maturation, receive excitatory input connections from hilar cells, and at the same time, start forming outgoing synapses with CA3 pyramidal cells [138]. The new GCs then proceed to form scarce excitatory connections with mature GCs [139]. This glutamatergic input together with back-propagating activation from CA3 pyramidal cells and hilar cell signals constitutes the only early source of excitatory inputs which is crucial for the survival of these cells [140]. Current hypothesis states that the new neurons act specifically on the mechanisms of pattern separation, decorrelating the new input from existing memory traces. Importantly, numerous environmental factors, like exercise, stress and antidepressants have been shown to affect the rate of neurogenesis in the SGZ of rodents [141,142]. 

As previously discussed, the existence of human adult hippocampal neurogenesis (AHN) has been a long theme of debate since it was reported, with evidence that support or reject it [102,143]. Despite the controversy, most evidence agrees that there is currently no reason to abandon the idea that, similar to what happens in rodent models, human adult-generated neurons make important functional contributions to neural plasticity and cognition across human lifespan [144].

### 3.3. Regulators of Neurogenesis

Several signals are key factors in the regulation of NSC proliferation, differentiation, migration and survival. These include morphogens, growth factors, neurotrophins, cytokines, neurotransmitters, extracellular matrix, cell-cell signaling molecules and systemic factors [145,146,147,148,149,150,151].

#### 3.3.1. Morphogens and Growth Factors

Fibroblast growth factor (FGF) signaling contribution ranges from regional patterning to the control of cortical size and neuronal fate. Wnt-dependent signaling on cortical neurogenesis are various and highly dependent on the cell stage and type [152,153]. Similarly to Wnts, bone morphogenic proteins are expressed dorsally in the midline and regulate regional patterning of the dorsal-most parts of the forebrain, but their impact on neurogenesis remains essentially unexplored [154,155]. *In vitro*, epidermal growth factor (EGF) was shown to be an important regulatory factor of adult NSCs [146].

#### 3.3.2. Neurotrophins

Neurotrophic factors such as brain-derived neurotrophic factor (BDNF), nerve growth factor (NGF), and neurotrophin-3 (NT-3) are known to be implicated in AHN [156]. Among these, BDNF is being intensively studied and has been shown to be involved in learning, memory and synaptic plasticity [157]. 

#### 3.3.3. Cytokines

Depending on the their type and mode of action, cytokines may have distinct roles in the brain and in NSC modulation: besides confering immune protection, by clearing the system of dead and/or damaged neurons, they can also exert harmful effects on NSC niches, leading to cell death [158,159,160].

#### 3.3.4. Neurotransmitters

Different neurotransmitters have been proposed as being regulators of different stages of the neurogenic process [149]. Dopamine has been linked to the modulation of cell proliferation [161]. Serotonin, however, depending on which receptor it acts, can either increase or decrease the proliferation of NSCs [162]. GABA has been shown to increase dendritic growth in neuroblasts migrating to the olfactory bulb [163]. Glutamate can influence both proliferation and neuronal commitment and can act as a positive regulator of neurogenesis [164].

#### 3.3.5. Extracellular Matrix

Cell behavior can be affected by the extracellular matrix in two main ways. One, by harboring growth factors or growth factor-binding proteins, and the other by cell–extracellular matrix interactions, which can directly regulate cell behavior through receptor-mediated signaling or by modulating the cellular response to growth factors [165]. 

#### 3.3.6. Cell-cell Signaling Molecules

The apical–basal polarity of NSCs is a decisive factor regarding symmetric versus asymmetric division, having an impact on cell proliferation. Lateral cues for spindle orientation, like occludins and epithelial (E)-cadherin coming from tight or adherens junctions, respectively, and other cytoskeletal components are overexpressed when cells are undergoing symmetrical cell division and downregulated during asymmetrical division [82,166].

#### 3.3.7. Systemic Factors

The proximity of adult NSCs to blood vessels, together with the cellular architecture of the niches, raises the possibility that the function of NSCs may be regulated by the balance of two independent forces, internal CNS-derived cues and external peripheral cues delivered in part by the circulatory system. Any external factor such as exercise, drugs, aging, among others, may change this delicate balance and, therefore, have an impact on NSC dynamics [167,168,169].

## 4. Cannabinoid Regulation of Neurogenesis

### 4.1. Cannabinoid Actions in Embryonic Neurogenesis

eCB signaling is involved in the regulation of several aspects of neural development, namely neural progenitor proliferation, lineage commitment, radial and tangential migration of pyramidal cortical neuron and interneurons, as well as axonal guidance, neuronal maturation and synaptogenesis. Both eCBs and cannabinoid receptors are present in the brain since early developmental periods [170,171]. eCBs, such as AEA and 2-AG, have been shown to be present from mid-gestation to adulthood. With low levels being present prenatally, AEA expression progressively increases during the perinatal period until reaching its maximum levels in the adult brain. 2-AG, in contrast, is highly expressed prenatally, displaying similar levels to those found in the adult brain [170]. Importantly, CB1Rs and CB2Rs have also been detected in the developing nervous system. In fact, embryonic stem cells have been shown to display both types of cannabinoid receptors [172]. CB1R expression levels increase during neuronal differentiation and gradually localize to developing axonal projections, whereas CB2Rs are more expressed in less committed cells and in microglia/macrophage lineages during development [171,173,174,175,176]. Both CB1R and CB2R activation were shown to boost, *in vitro*, mouse neural stem and precursor cell proliferation [175,177,178,179]. eCB signaling is active in ventricular/subventricular zones from embryonic day 12 onwards, being responsible for controlling the proliferation of pyramidal cell progenitors [174,180]. In addition, AEA, through CB1R activation, was shown to enhance NSC differentiation into neurons, namely the differentiation into corticospinal motor neurons, with CB1R basal activity playing a role in the initial development of dendrites and, indirectly, in axon initial segment development [177,181,182].

Cannabinoids have an essential role in creating the architecture and wiring of the brain. Through CB1R signaling, they regulate radial and tangential migration of post-mitotic neurons. Indeed, CB1R signaling is involved in the regulation of long-distance migration of late-gestational GABAergic interneurons, as well as radial migration of immature pyramidal cells [174,183,184]. In addition to this, axonal growth cones of cortical GABAergic interneurons, which are enriched with CB1Rs, are activated by eCBs, acting as axon guidance cues that trigger CB1R internalization and elimination from filopodia, thus contributing to pathfinding [173,185]. In fact, when these receptors are antagonized or CB1R gene is knocked down, the resulting lack of CB1R signaling impairs axon pathfinding and fasciculation in zebrafish embryos [186]. Long-range axonal connectivity is modulated by CB1R signaling through regulation of corticofugal axon navigation and fasciculation during corticogenesis [174,187]. 

During gestation, phyto- and synthetic cannabinoids can pass the blood-placental barrier, consequently affecting fetal brain development [188]. As this is a critical period, ECS manipulation may have an impact on cognition and behavior [189]. Indeed, children born from women who consumed marijuana during pregnancy display cognitive deficits, namely in executive function, working memory tasks, sustained attention and learning, as well as psychiatric disorders [190,191,192,193,194,195]. However, it is important to mention that cognitive deficits derived from heavy marijuana use during the prenatal period alone were shown to be less severe than the ones resulting from the combined effect of marijuana with other drugs of abuse, namely nicotine and alcohol [192,196]. Prenatal exposure to marijuana was also negatively associated with performance in tasks that required visual memory, analysis and integration in adolescents [197]. Accordingly, prenatal exposure to WIN 55,212-2 (a non-selective cannabinoid receptor agonist) in rodents was shown to impair tangential and radial migration of post-mitotic neurons in the dorsal pallium [198]. Moreover, Δ^9^-THC administration in mouse models was shown to induce rapid neuronal remodeling, such as retraction of neurites and axonal growth cones, elevated neuronal rigidity and reshaping of somatodendritic morphology [184,199]. In addition to this, in rat models, prenatal exposure to WIN 55,212-2 or Δ^9^-THC induced long-lasting learning and memory impairments in the adult offspring, as well as permanent alterations in hippocampal long-term potentiation, hippocampal glutamate release and cortical glutamatergic neurotransmission [200,201]. Although maternal consumption of AEA did not affect CB1R expression in the brains of pups, enhancing eCB signaling led to subtle behavioral deficits in the adult offspring, revealed by reduced cocaine-conditioned preference test, increased depressive-like behaviors and impaired working memory [202,203]. Moreover, Δ^9^-THC administration during pregnancy was shown to interfere with corticospinal connectivity and to produce long-lasting alterations in the fine motor performance of the adult offspring [204]. Due to CB1R-mediated regulation of both glutamatergic and GABAergic neuronal development, Δ^9^-THC administration in this period also increased seizure susceptibility [204]. In addition, Δ^9^-THC was also reported to trigger the stress-activated protein kinase c-jun N-terminal kinase and the pro-apoptotic protease caspase-3 in *in vitro* cerebral cortical slices, obtained from neonatal rat brains, an effect that was not observed in adult rat brain slices, which demonstrates the brain vulnerability during the perinatal period [205]. Furthermore, early Δ^9^-THC exposure during brain development was also shown to compromise astroglial cells since GFAP and glutamine synthetase expression was reduced [206]. 

The effects on brain function and behavior, mediated by cannabinoid signaling modulation during neurogenesis, are also dependent on cannabinoid concentrations. For instance, low concentrations of Δ^9^-THC and AEA did not affect neuronal and dopaminergic (DA) maturation, with AEA only enhancing the frequency of synaptic activity. In contrast, higher doses of these CB1R agonists reduced neuronal function by decreasing synaptic activity and ion currents [207]. 

These findings show the importance of eCBs as key regulatory factors of brain structuring and wiring, warning, at the same time, for the impact that exogenous cannabinoids may have on cognition and behavior when administered during this critical period of neurodevelopment.

### 4.2. Cannabinoid Actions in Postnatal Neurogenesis 

In addition to their modulatory role of embryonic development, discussed above, there is considerable evidence to suggest that both endogenous and exogenous cannabinoids are able to regulate postnatal neurogenesis by acting on distinct steps of NSC regulation, although the effects can vary considerably according to the cannabinoid, dose and protocol of administration [208,209,210,211]. In this section we focus on emerging literature that proposes cannabinoids as regulatory agents of NSC proliferation and maturation in the SVZ and SGZ of the adult brain. Importantly, cannabinoid signaling influences the identity and cellular features of adult NSCs because its expression changes during differentiation and its mechanisms of action promote the activation of proliferative and/or pro-survival cascades, which are essential in the regulation of cell cycle [210,212]. 

Several studies have provided compelling evidence linking cannabinoids and NSC regulation in the adult brain [210,213,214]. Notably, more attention has been given to the actions of the major cannabinoid receptors on adult NSCs. 

CB1R contribution to adult neurogenesis has been shown to be fairly robust [180,208,213]. Indeed, early studies indicated that CB1R knockout (KO), in mice, results in impaired neurogenesis, suggesting a regulatory role of CB1Rs in adult neurogenesis [213]. Moreover, the use of ACEA (CB1R selective agonist) was shown to promote mice neural precursor differentiation towards a neuronal lineage, suggesting that CB1R activation may represent a pro-neuronal differentiation signal [177]. Similarly, CB1R activation (with R-m-AEA) was shown to induce proliferation, self-renewal and neuronal differentiation in mouse neonatal subventricular cell cultures [215]. Interestingly, treatment with a CB1R antagonist AM251 abolishes an exercise-induced increase of hippocampal cell proliferation, indicating that endogenous cannabinoid signaling is required for exercise-mediated NSC proliferation [216]. Moreover, a recent study elegantly shows that activation of CB1Rs within the NSC lineage itself is essential to control neurogenesis in adult mice by regulating NSC pool, dendritic morphology, activity-dependent plasticity and behavior [217]. In line with this, numerous studies have found CBD to increase both NSC proliferation and overall neurogenesis, with some indication that it may do so through interactions with CB1Rs, CB2Rs and PPARγ [218,219,220,221]. 

In accordance, CB2R activation with selective agonists was also shown to influence the proliferation, differentiation and survival of adult NSCs [222,223]. In one study, a CB2R KO was shown to have reduced self-renewal capacity of murine embryonic cortical NSCs, while activation with CB2R agonists increased primary neurosphere generation and neural progenitor self-renewal *in vitro* [175]. In another study, the administration of HU-308 (a CB2R selective agonist) was shown to induce the proliferation of NSCs via phosphatidylinositol 3-kinase (PI3K)/Akt/mammalian target of rapamycin (mTOR)-dependent signaling both *in vitro* and *in vivo* [224]. Moreover, treatment with a cannabinoid receptor agonist (WIN 55,212-2) or with a CB2R selective agonist (JWH-133) in an *in vivo* study showed an increase in SVZ NSC proliferation, this effect being more pronounced in aged mice [225]. Another example is found in the use CB2R agonist AM1241, which has been shown to promote the proliferation/differentiation of primary human NSCs *in vitro*, as well as a reduction in astroglyosis and gliogenesis *in vivo*, suggesting a neuroprotective role of CB2Rs [226]. 

Several studies using technology to limit the availability of eCBs, by targeting endogenous cannabinoid metabolism, also show compelling evidence linking cannabinoids and adult NSC regulation. For example, chronic inhibition of the enzyme responsible for the production of 2-AG (DAGL) was shown to abolish cell proliferation, while inhibition of FAAH, the enzyme responsible for AEA hydrolysis, promoted an increase in cell proliferation in the mouse SVZ [225]. Supporting this, it was observed that knockdown of FAAH promoted a substantial increase in cell proliferation in the DG of adult mice [180]. Another study showed that complete knockdown of the DAGLα subtype enzyme promoted a general reduction of 2-AG and AEA levels, with a concomitant decrease in cell proliferation and reduction in the expression of immature neurons in the mouse hippocampus, suggesting the importance of basal eCB tone in maintaining NSC homeostasis [227].

Apart from affecting the production of new neurons in the adult brain, existing evidence shows that cannabinoids are also modulators of astro- and oligodendrogenesis in the early postnatal brain [214,228,229]. In fact, it was observed that postnatal CB1R activation was able to promote astroglial differentiation of mouse neural progenitor cells *in vitro* and *in vivo* [228,230]. Moreover, both CB1Rs and CB2Rs were detected in oligodendrocyte progenitor cells (OPCs) and 2-AG was reported to enhance early OPC proliferation [231,232]. Additionally, either non-selective activation of cannabinoid receptors by WIN 55,212-2 or selective activation of CB1Rs or CB2Rs stimulates OPC differentiation into oligodendrocytes [229,233,234]. In fact, pharmacological activation of CB1Rs and CB2Rs was shown to enhance the expression of myelin basic protein in the subcortical white matter and to promote oligodendrogenesis in the SVZ of adult animals [235]. Evidence suggests that activation of CB1Rs and CB2Rs was found to enhance the survival of OPCs *in vitro* via activation of ERK and PI3-AKT signaling pathways [178,231]. Moreover, the administration of WIN 55,212-2 was found to stimulate the survival of adult-born OPC in a viral encephalitis-induced animal model, depicting the role of eCB signaling in modulating the survival and generation of glial cells during postnatal stages [236].

Further data supporting the role of cannabinoids in adult NSCs shows that cannabinoids can interact with other neuromodulatory signals. Indeed, recent evidence shows that CB1R agonist ACEA can target fascin, an actin-bundling protein, and interact with protein kinase C (PKC) signaling pathway promoting the migration of neuroblasts [237]. Moreover, administration of CBC, a non-THC phytocannabinoid, to adult neural precursor cells extracted from brains of eight week-old mice was found to promote cell survival during differentiation and to dampen astroglial differentiation through the involvement of ERK, ATP and adenosine signaling cascades [238]. CB1Rs and CB2Rs were also shown to cooperate with EGF receptors in the regulation of NSC expansion [239]. In the same way, CB1Rs have been shown to activate FGF receptors in order to promote axonal growth in rat cerebellar granule neurons [240]. Further evidence has shown that pharmacological blockade of CB1Rs and/or CB2Rs leads to a decrease in NSC proliferation, accompanied by a decline in the expansion of mouse brain-derived neurospheres, and that interleukin 1 (IL-1) signaling pathway was involved in this process, emphasizing the neuroimmune interactions of cannabinoid signaling [241].

Overall, most evidence shows that genetic ablation of cannabinoid receptors or treatment with CB1R and CB2R selective antagonists leads to a decrease in NSC proliferation in the hippocampus and SVZ [175,213,217]. By contrast, either direct activation of cannabinoid receptors using synthetic agonists and/or an indirect approach aiming at increasing eCBs, by inhibiting their degrading enzymes, stimulates the formation and maturation of new neurons in both adult niches [213,242,243]. 

The complexity/variability of these findings also illustrates that study design, animal species, strain, gender and/or compound selectivity and dosage are very important when studying the effects of cannabinoids in NSC regulation. Moreover, data disparity of many studies involving adult NSCs may in part be explained by the varying properties of each NSC population and the heterogeneity within each NSC pool, which suggests that eCB signals, acting in a particular spatial and temporal manner, could differentially affect the proliferative capacity of NSCs, limiting the lineage specification of succeeding progenies. In fact, there are divergences between the two niche microenvironments regarding the molecules that regulate morphogenesis, rates of division, self-renewal and survival which may account for the observed differences [244,245].

## 5. Role of Cannabinoids in Neurogenesis and Pathophysiology

Neurological and mental disorders comprise a broad range of disabling conditions with different phenotypic outputs. Some symptoms of these disorders may arise from early impairment in neural development or aberrant adult neurogenesis [246]. In fact, alterations in the mechanisms behind NSC regulation in the embryonic and adult brain have been reported in both patients and animal models of AD, Parkinson’s disease (PD), MS, epilepsy and mood disorders [247,248,249,250,251]. 

Despite the extensive evidence suggesting that both exogenous and endogenous cannabinoids regulate NSC proliferation, which in turn may be affected by disease loading, the link between NSCs, cannabinoids and brain disorders remains to be established. This unclear vision may arise from the poor/varied design of pre-clinical studies, the lack of post-mortem analyses of brains from patients with neurological disorders as well as the lack of clinical studies involving cannabinoid usage. However, several data suggest that manipulation of cannabinoid signaling promotes a fine-tuned regulation of NSCs, which is resultant from their anti-inflammatory and antioxidant properties. Therefore, eCBs may have an impact in delaying, preventing or restoring some neural deficits in animal models that mimic some features of neurodegenerative and psychiatric disorders, therefore, constituting potential therapeutic targets for neuroregeneration.

### 5.1. Cannabinoids and Neuroprotection 

Brain damage and neurodegeneration are leading causes of long-term disability, disease burden and mortality worldwide [252]. They are characterized by the progressive loss of specific neuronal subpopulations in the CNS, with different clinical features being exhibited and can result from the natural process of brain aging or brain injury/trauma. Available treatments usually act on transient symptomatic relief, being poorly active at fighting the cellular events occurring as brain damage advances [253]. Therefore, it is essential to seek for molecules with disease-modifying activity that take into consideration the mechanisms that underlie disease progression and enable the repair of neuronal loss. 

The neuroprotective potential of ECS-targeting compounds (e.g., cannabinoid agonists or antagonists, inhibitors of eCB degradation or biosynthesis or other modulators) has been extensively investigated over the last 20 years [253]. This neuroprotective nature of cannabinoids arises not only from their pleiotropic profile, i.e., the capacity to interact with neuromodulatory systems not directly related with the eCB signaling, but also from the presence of these targets in key cellular components of the CNS (i.e., neurons, astrocytes, microglia, oligodendrocytes and neural progenitor cells) and brain structures like the blood–brain barrier [58]. These features then translate into an ability to restore the CNS to a physiological homeostatic state after an acute or chronic perturbation, by fighting an array of cellular processes caused by brain insults or damage, like excitotoxicity, increased state of neuroinflammation, oxidative stress and protein aggregation, as well as other processes that interfere with neuronal homeostasis and integrity [253]. Given the relationship between all these pathological cellular hallmarks, the protection of CNS components must act on all or most of these targets in a multifaceted manner. Therefore, cannabinoids are valuable candidates for this strategy.

Excessive release of glutamate generates accumulation of toxic concentrations of intracellular calcium and oxygen free radicals, resulting in excitotoxicity, a process common to many brain disorders which often leads to neuronal death [254]. A number of observations indicate the ability of cannabinoids to control glutamate release through the activation of CB1Rs and have revealed a crucial role of this receptor in excitotoxicity control, BDNF being a key mediator of this process [43,255]. Others have also demonstrated that cannabinoid-mediated neuroprotection against excitotoxicity relies on CB1R and CB2R modulation in glial cells [256]. Moreover, inhibition of eCB uptake with UCM707 was shown to have a protective role against AMPA-induced excitotoxicity through activation of CB1Rs, CB2Rs and PPARγs [257].

While the mechanisms of neuroprotection aimed at regulating glutamate homeostasis are mainly due to CB1R actions, the anti-inflammatory effects of cannabinoid-mediated protection are mostly attributed to the modulation of CB2Rs. Hence, the involvement of the ECS, particularly of CB2Rs, in reducing local or systemic inflammatory events is a crucial part of cannabinoid-mediated neuroprotection. Neuroinflammation is the process by which the release of cytotoxic agents and cell death occurs after acute or chronic CNS damage [258]. The regulation by cannabinoids of mechanisms like modulation of immune responses and the release of inflammatory mediators was extensively reviewed in [259]. For instance, mouse microglial cell cultures treated with CB2R selective agonist JWH015 showed a reduction in interferon-γ (IFN-γ)-induced upregulation of CD40 expression, which lead to a decrease in the production of proinflammatory cytokines and an enhancement of amyloid-β (Aβ) phagocytosis [260]. Moreover, administration of CBD and WIN 55,212-2 was shown to reduce the levels of proinflammatory cytokine IL-6 derived from Aβ exposure [261]. Further studies have reported that oral administration of JWH-133, a potent CB2R agonist, decreased microglial activation, proinflammatory factors COX-2 and tumor necrosis factor α (TNF-α) mRNA expression and the cortical levels of Aβ in a transgenic mouse model of AD [262]. In the context of aging, which is closely related with an increase in neuroinflammation, aged rats administered with WIN 55,212-2 showed a reduced number of activated microglia in the hippocampus and DG. Furthermore, the same treatment was found to decrease the mRNA levels of the IL-6, as well as the protein levels of the inflammatory factors TNF-α and IL-1β [263].

In like manner, cannabinoid receptor ligands are often reported as having antioxidant properties because they are able to fight oxidative stress and reduce reactive oxygen species (ROS) load, processes intimately associated with excitotoxicity and neuroinflammation. For example, Δ^9^-THC exposure was shown to rescue the pharmacologically-induced inhibition of mitochondrial function, ubiquitin proteasome and production of free radicals in a human neuroblastoma cell line [264]. Moreover, treatment with CBD promoted potent antioxidant actions in both *in vitro* and *in vivo* studies by reducing ROS burden [265,266].

In addition to the benefits resulting from direct activation of the ECS, the ability of eCBs to interact with other molecules/systems can also provide neuroprotective actions. In fact, cannabinoid-mediated neuroprotective effects also result from interactions with transcription factors, neurotrophic factors, receptors of the PPAR family or with elements of other transmission systems, like the serotonin 5-HT1A receptors or adenosine A2A receptors [267,268,269,270,271,272].

Importantly, the ECS has been shown to be involved in the neuroprotection of NSCs and their resulting progeny. In fact, CB1Rs were shown to be involved in the excitotoxicity-induced neural progenitor cell proliferation and neuronal differentiation [273]. Moreover, activation of CB2Rs was shown to increase the survival of NSCs and progenitor cells, as well as to rescue impaired hippocampal neurogenesis caused by chronic insult by HIV-1 neurotoxic protein gp120 [226]. More recently, activation of GPR55 has demonstrated a neuro-immune regulatory role of human and murine NSCs by protecting them against chronic inflammation *in vitro* (with IL-1β treatment) and *in vivo* (with chronic LPS administration) [274].

Despite the neuroprotective actions associated with the ECS modulation, cannabinoid-induced neurotoxic effects have also been reported [252]. This inconsistency may be due to numerous factors, such as the severity and timing of the pathologically-induced insult and, the kind of pharmacological intervention implemented. The dosage and timing of administration of cannabinoids are also of great importance, given that high exposure usually induces neuronal cell death [275]. Moreover, different cannabinoids have different mechanisms of action depending on the type of receptors they modulate and the corresponding signaling pathways. 

In the next sections, illustrative examples of ECS system modulation in disease context will be described in more detail (Supplementary Table S1).

### 5.2. Cannabinoids and Brain Disorders 

#### 5.2.1. Alzheimer’s Disease

Late-onset AD (≥65 years-old) is responsible for about 70% of all cases of dementia [276]. The disease is characterized by a number of Aβ oligomerization and aggregation-induced neurotoxic mechanisms, including tau hyperphosphorylation, neuroinflammation with reactive gliosis (astro and microgliosis), excitotoxicity and oxidative damage [277,278,279]. These changes, which primarily impact on the cortex and the hippocampal formation, result in disrupted synaptic plasticity, loss of synaptic function, namely cholinergic, and cell death [277,278,279]. Despite being one of the most debilitating neurodegenerative disorders, present available treatment options have failed to stop or even significantly delay disease progression [278]. Due to its broad range of actions at the PNS and CNS, modulation of the ECS has been extensively discussed as a potential multimodal disease-modifying therapy for the management of complex multifactorial conditions such as AD (reviewed in [7,280,281]). 

AD patients classically show a slow-progressing cognitive impairment, notably affecting short- and long-term memory performance, which are highly dependent on hippocampal function [277,282,283]. However, clinical evidence assessing the potential benefit of cannabinoids on cognitive decline is currently lacking [284]. Nonetheless, a few patient trials have focused on the use of the THC-based pharmaceutical formulation dronabinol (Marinol^®^) for relieving neuropsychiatric symptoms shown by most demented individuals, denoting a decrease in agitation, nocturnal motor activity and aggressive behaviors, as well as an increase in body weight [285,286,287,288].

Changes in several components of the ECS have been described in AD post-mortem samples and animal models of the disease. Despite contradicting results, these changes appear to be dependent on the type of cells, but also on disease stage, and Aβ aggregation state [7,289]. In this regard, an initial increase of CB1Rs is proposed to occur, followed by a downregulation at later stages [290]. Interestingly, CB2Rs are overexpressed in microglia surrounding neuritic plaques, yet decreased in neurons [50,291,292]. Increased 2-AG signaling is also observed around Aβ plaques in late-AD, in association with diminished MAGL activity and enhanced DAGL expression [293,294]. In addition, augmented FAAH activity and expression can be denoted in astrocytes near plaques, with subsequent reduction of AEA [291]. 

How the regenerative capability of the adult brain is modulated in aging and AD is still a matter of debate, with variable results from *in vitro* and *in vivo* (both pharmacological and transgenic) animal models and post-mortem human samples [295]. However, most evidence points to a depletion of proliferation, differentiation and survival of NSCs, as well as compromised morphology and maturation of GCs in the DG of mouse models of AD [296,297,298,299,300,301,302]. Moreover, as for the ECS, alterations in AHN are suggested to be dependent on disease progression and Aβ conformation, being initially elevated as an attempt to compensate for neurotoxicity and cell death, yet fading at later disease stages [303,304].

The use of different models of AD shows extensive promise regarding the neuroprotective role of cannabinoids. A relevant function of these compounds in reducing amyloid burden and associated neurotoxicity has been suggested *in vitro*. Δ^9^-THC is a competitive inhibitor of acetylcholinesterase (AChE) activity, potentially preventing AChE-induced Aβ aggregation [305]. Both 2-AG and AEA, as well as CB1R/CB2R (WIN 55,212-2 and HU-210), and CB2R (JWH-133, JWH-015) agonists, have been shown to prevent Aβ-induced toxicity, namely by promoting microglia-mediated Aβ clearance [260,292,306,307,308]. Similar effects of CBD or PPARγ activation were also observed, demonstrating enhanced cell survival, decreased oxidative stress, and regulation of Aβ production and clearance [309,310,311,312,313]. 

Furthermore, many authors have focused on the cannabinoid-mediated anti-inflammatory actions, mainly using transgenic mouse models of AD or models obtained by intracerebral injections of Aβ to mice and rats. In these models, neuroinflammation and reactive gliosis around neuritic plaques was shown to be effectively down-regulated by MAGL inhibition, WIN 55,212-2, CB1R agonist ACEA, CB2R agonists MDA7 and JWH-133, CBD and THC+CBD [218,261,262,292,314,315,316,317,318,319,320]. Besides these effects, various studies using the same compounds and AD models have demonstrated learning and memory improvements, in tasks that significantly rely on hippocampal function, although some authors report no effect on Aβ load [261,262,292,293,314,315,316,317,318,319,321,322,323]. In line with this, Δ^9^-THC and MAGL inactivation have been shown to decrease the occurrence of neuritic plaques in a transgenic mouse model carrying five mutations related to familial AD (5xFAD) [324,325].

It is still unclear whether cannabinoids might have a positive impact on AD pathology, partly through regulation of AHN, yet a few common pathways may be mentioned. Some of the aforementioned anti-inflammatory and neuroprotective actions of cannabinoids have been linked to the inhibition of glycogen synthase kinase 3β (GSK-3β) overactivation, which is known to promote tau hyperphosphorylation and Aβ production, and is a negative regulator of AHN [218,262,319,320,326,327,328,329]. In fact, CBD has been found to suppress reactive gliosis and rescue neurogenesis in the DG of rats injected with Aβ1-42 in a PPARγ-dependent manner, likely through inactivation of GSK-3β and subsequent rescue of Wnt/β-catenin pathway, an important regulator of AHN [218,320,330,331]. Administration of MDA7, a potent CB2R selective agonist, to AD transgenic amyloid precursor protein/presenilin 1 (APP/PS1) mice was observed to reduce microgliosis, promote Aβ clearance, restore memory performance, synaptic plasticity and Sox2 expression, a transcription factor expressed by NSCs in the DG [318,332]. Likewise, AEA was shown to enhance Notch-1 signaling, a known modulator of AHN, which was impaired by Aβ in cultured neurons [331,333]. 

A noteworthy parallelism can be made between a dose-dependent effect of Δ^9^-THC on memory and neurogenesis, where low concentrations of the compound appear to improve memory function and promote AHN, while higher doses seem detrimental (recently reviewed in [334]). Additionally, given the elevated density of cannabinoid receptors in the hippocampus and the relevance of the ECS in regulating AHN, it becomes evident that the therapeutic value of cannabinoids for AD pathology may rely on restoring aberrant neurogenesis [335].

Although preclinical evidence has been supportive of the administration of cannabinoids to ameliorate AD pathology, a clinical benefit remains to be assessed due to the limited number of clinical trials, with short duration and low number of subjects, that fail to evaluate cognitive parameters, as well as biomarkers of neurodegeneration [281,336]. In addition, future studies are needed to assess long-term safety and effectiveness of natural and synthetic cannabinoids, specifically in older individuals with AD [280,336]. Importantly, there is a pressing need for a better comprehension of the underlying mechanisms concerning the interaction of cannabinoids in AD and hippocampal NSC modulation, namely using specific neurogenic markers. 

#### 5.2.2. Parkinson’s Disease

PD is characterized by a progressive degeneration and subsequent loss of DA neurons in the substantia nigra pars compacta (SN) [337]. These neurons compose the brain motor system, being responsible for the initiation of movement and the reward pathway, by innervation of the striatum [338]. The presence of Lewy bodies, which are mainly formed of alpha-synuclein (αSyn) aggregates, have been identified as a prerequisite for the post-mortem diagnosis of both the pre-symptomatic and symptomatic phases of the pathological process underlying PD. These aggregates have been identified as belonging to two distinct categories, the brainstem- and the cortical-derived, with the latter type being more strongly immunoreactive for αSyn [339,340,341]. Symptomatically, PD is a progressive movement disorder that causes muscle rigidity, tremors, bradykinesia and shuffling gait [342]. It can also cause dementia, especially in advanced stages [338]. The motor dysfunctions, which are the main feature in PD, become symptomatic when ≈60% of neurons are already lost [341]. Although in the vast majority of cases PD is idiopathic, epidemiological evidence suggests that environmental toxins such as pesticides increase PD risk [340,343]. However, in some cases, PD is associated with inherited mutations in PD-related genes, such as α-Synuclein (*SNCA*), parkin (*PARKIN)*, PTEN-induced putative kinase protein 1 (*PINK1*), ubiquitin carboxyl-terminal esterase L1 (*UCH-L1*) and leucine-rich repeat kinase 2 (*LRRK2*) [344]. Current therapeutics for PD rely mostly on the use of pharmacological agents, mainly through the use of L-DOPA, a dopamine precursor [345]. These are often used to improve motor symptomatology of PD patients, rendering no effective cure for PD. Therefore, the development of new strategies has been the focus of current PD research. 

Targeting the ECS may prove as an alternative therapy to improve motor symptoms, as PD patients reported an amelioration in bradykinesia, accompanied by a reduction in muscle rigidity and tremors after cannabinoid intake [346]. These reports are supported by three major pieces of evidence. First, the basal ganglia and cerebellum, brain areas responsible for the control of movement, which are highly affected in PD, express CB1R, CB2R and TRPV1R [347,348,349]. Second, it has been shown that motor activity is repressed by the strong inhibitory action promoted by a variety of cannabinoids, which are responsible to fine-tune the activity of various classical neurotransmitters [350,351,352,353]. Finally, evidence shows that ECS signaling is altered in the basal ganglia of humans and in animal models of PD [354,355,356]. These clinical-based evidence are supported by robust pre-clinical data which indicates that the ECS has a neuroprotective role in PD [357,358]. One study, using a rotenone-induced rat model of PD supplemented with β-caryophyllene (BCP), a naturally occurring CB2R agonist, showed a decrease in the levels of proinflammatory cytokines and inflammatory mediators. These results were further supported by an increase in tyrosine hydroxylase immunoreactivity, which illustrated the rescue of the DA neurons and a reduction in the activation of glial cells [359].

In human PD post-mortem studies, the endogenous pool of adult NSCs was shown to be significantly affected, specifically in the SGZ, suggesting a potential effect of dopamine on NSC proliferation and survival, as reviewed by [161,360]. New evidence suggests that adult NSCs are also impaired in animal models of PD, supporting data from human studies. Although the precise mechanisms and effects of these changes are not yet fully understood, αSyn and aging were shown to decrease adult neurogenesis throughout the several stages of PD [361,362,363]. 

One of the earliest stages of PD is characterized by a non-motor symptom, namely hyposmia or anosmia, which is the loss of the sense of smell, being reported in 90% of patients [364]. Alterations in olfaction in PD seem to be related with changes in central olfactory processing, which could be explained by αSyn pathology being present in the olfactory bulb long before Lewy bodies are detected in the SN [341]. In fact, neurogenesis in the SVZ and olfactory bulb was shown to be impaired in a transgenic mouse model expressing the human αSyn carrying the A30P mutation, where significantly fewer newly generated neurons were observed in the olfactory bulb [365]. Other studies, using the Parkinson 6-hydroxydopamine (6-OHDA) rat model have shown that 6-OHDA induced SN DA degeneration and had a major impact on SGZ neurogenesis [366,367]. These reports suggest that dopamine depletion reduces NSC proliferation and consequently adult neurogenesis [368].

Therefore, potentiating the intrinsic pool of NSCs has been proposed as an alternative potential PD therapy. Several studies, with contradictory findings, have been focusing on stimulating the production of DA neurons from the SVZ and SGZ. In fact, it was shown that the administration of D1 and D3 receptor agonists in the 6-OHDA rat model, produced an increase in SGZ and SVZ cell proliferation, respectively [367,369]. Adding to that, a D3 receptor agonist also increased DA newborn neurons in the SN, leading to the improvement of motor impairments [369]. Another study failed to induce SN DA neurogenesis using the D2/D3 receptor agonist pramiprexole, however it promoted olfactory bulb DA neurogenesis [370]. Another growing strategy is the use of human pluripotent stem cells to induce DA differentiation. These can be derived from early pre-implantation embryos (embryonic stem cells, ESCs) or by reprogramming adult somatic cells (induced pluripotent stem cells, iPSCs), and then differentiated into midbrain DA neurons using recently developed protocols [371,372]. However, brain implantation of these cells requires invasive surgical techniques and generates side effects (e.g., graft-induced dyskinesias) with signs of disease-related pathology in the transplanted cells being visible years after implantation [373,374,375]. A cannabinoid induction of DA differentiation from NSCs is still under debate [207,215]. One recent study compared AEA with Δ^9^-THC and concluded that, using higher doses of these compounds, the functional maturation and DA specification from human cord blood-derived iPSCs was significantly compromised [207]. 

To conclude, in PD, it has been shown in several studies with both human post-mortem samples and animal models that both neurogenic niches are significantly impaired by αSyn pathology or DA depletion [360,361]. Replenishing the DA neuronal loss has been proving a challenge to the scientific community, whether by using the endogenous pool of NSCs, or by targeting the ECS, to promote neurogenesis at specific and selective timepoints. The ECS may still be applied clinically in order to ameliorate PD symptomatology due to the aforementioned neuroprotective properties of cannabinoids and therefore improving the quality of life of PD patients [358,376,377].

#### 5.2.3. Multiple Sclerosis

MS is one of the most recurrent disorders of the CNS. Despite its unknown etiology, an immune response and consequent infiltration of immune cells into the CNS together with demyelinating events culminates in oligodendrocyte loss and neuronal degeneration. Some of the resulting symptoms are spasticity, tremors, ataxia, bladder dysfunction and neuropathic pain, with a high impairment of the quality of life of the patient [378,379,380]. MS patients that consumed cannabis reported relief regarding several of these symptoms, highlighting a possible role for cannabinoids in MS [380,381,382,383,384,385]. Furthermore, the neuroprotective effects of these molecules in MS has been thoroughly described in the literature, since they are able to diminish oligodendrocyte death and increase remyelination whilst having an anti-inflammatory role [231,386,387]. Changes in eCB levels and also in the levels of its receptors and degrading enzymes, FAAH and MAGL, were observed both in blood and post-mortem brain samples of MS patients and in animal models of MS, such as the experimental autoimmune encephalomyelitis (EAE) model, in different stages of disease [379,383,388,389,390,391,392,393]. 

Studies using EAE-induced mice where CB1Rs and CB2Rs were genetically deleted or pharmacologically inhibited show elevated neurodegeneration and poorer anti-inflammatory responses accompanied by an increase in EAE severity and motor impairment [379,386,394,395,396]. The potential targets for ECS modulation in MS, namely the degrading enzymes and transporters which actively participate in this mechanism by controlling the levels of eCBs have been a matter of study [380,397,398,399]. However, the exact mechanisms for the actions of cannabinoids in MS are still not totally known.

Using the Theiler murine encephalomyelitis virus-induced demyelinating disease (TMEV-IDD) mice, it was possible to observe that the modulation of the ECS by inhibition of AEA uptake resulted in an improvement of motor performance, reduction of microglial/macrophage activation and proinflammatory cytokine release, accompanied by an increase in eCB signaling [400]. 

Recently, it has been shown that CBD attenuates EAE pathology by activating anti-inflammatory myeloid-derived suppressor cells in the periphery, or by modulating the increase of anti-inflammatory and decrease of proinflammatory cytokines, both *in vivo* and *in vitro* [401,402]. Additionally, treatment with WIN 55,212-2, a CB1R/CB2R non-selective agonist, was found to attenuate the interaction between leukocytes and endothelial cells, which is necessary for immune cells to infiltrate the CNS, therefore inhibiting infiltration. Moreover, by using selective CB1R and CB2R antagonists, it was observed that the effect on leukocyte trafficking exerted by cannabinoids is triggered by CB2R activation [403,404]. Furthermore, as it is intimately-related with immune responses, CB2R selective activation was shown to improve EAE phenotype by decreasing disease severity and incidence [405]. Additionally, the accumulation of CD4^+^ T cells in the brain and spinal cord is also decreased in these animals, with a response depending on the time of administration, at an early or late timepoint of disease course [405]. 

Throughout the years, numerous clinical trials worldwide have been using cannabis-based drugs in an attempt to treat MS symptoms. Three main active principles derived from cannabis formulate these drugs: dronabinol (Marinol^®^), a synthetic isomer of Δ^9^-THC, nabilone (Cesamet^®^), a synthetic analogue of Δ^9^-THC and nabiximols (Sativex^®^), a 1:1 mix of Δ^9^-THC and CBD [380]. Sativex^®^ consists of an oral spray approved in some European countries and Canada for the treatment of spasticity and pain relief in MS patients, with mild-to-moderate side effects that occur scarcely in patients [379,380,406,407,408,409,410,411,412,413,414,415,416]. Nevertheless, no trial has shown a slower disease progression in MS patients prescribed with THC-based drugs and, hence, there are still no evidence of its neuroprotective effect in humans [379,417,418]. 

Concomitantly, in MS there is myelin damage and oligodendrocyte loss, and thus, finding new mechanisms to promote both re-myelination and OPC differentiation, either from precursors present in the brain parenchyma or derived from SVZ NSCs is crucial, since OPCs are able to migrate and partially remyelinate lesioned areas [249,419,420,421]. Cells from the oligodendroglial lineage are directly modulated by cannabinoids in distinct maturation stages, ranging from the regulation of OPC survival, proliferation, migration and differentiation to the modulation of mature oligodendrocyte survival and myelinating capacity [422,423]. For instance, it has been observed that OPCs express CB1Rs and CB2Rs and that cannabinoids promote OPC survival and oligodendrocyte differentiation through the PI3K/Akt/mTORC1 signaling pathway, which is known to participate in the myelination process [229,231,422,424,425,426,427,428,429]. CBD has also been shown to be a modulator of this pathway and its administration to EAE mice was shown to decrease the infiltration of inflammatory immune cells into the CNS and to promote the phosphorylation of PI3K, Akt and mTOR, together with the inhibition of MAPK signaling pathway, leading to an anti-inflammatory response and neuronal survival [423]. 

Although several studies have looked at the relationship between cannabinoids and oligodendrocyte differentiation under inflammatory-demyelinating conditions, there is still a major gap concerning the ability of SVZ-derived NSCs to be modulated by cannabinoids and differentiate into OPCs, which could be useful for MS therapeutics and should be addressed in future studies [235].

#### 5.2.4. Epilepsy

Epilepsy is a neurological disorder characterized by a persistent predisposition to generate epileptic seizures which are often associated with neurobiological, cognitive, psychological and social consequences [430,431]. An epileptic seizure can be defined as an abnormal excessive and/or synchronous neuronal activity in the brain, which instigates a transient behavioral alteration, comprising a myriad of signs and symptoms (such as loss of awareness, stiffening, among others) [430]. Although most patients have idiopathic epilepsy, seizures can be induced by lesions or insults that impact normal brain function and activity. The main causes for epilepsy include lesions or structural alterations, such as stroke, tumor, traumatic brain injury, infectious diseases, metabolic alterations, autoimmune diseases and genetic mutations [430,432]. More than 500 genes have been linked to epilepsy, including 84 genes that directly cause epilepsies or syndromes with epilepsy as one of their core symptoms [433]. 

Epileptogenesis is the multifactorial process that underlies the development of spontaneous seizures. It occurs before and persists beyond the first unprovoked episode and can progress over several years in humans [430,432,434]. The mechanisms behind this process are still not completely understood but include a widespread of alterations in both neuronal and non-neuronal cells, leading to molecular and structural changes that result in the dysfunction of neuronal circuits [430,434,435]. The main alteration is an imbalance between excitation and inhibition in the neuronal circuits, through an increase in excitatory neurotransmission, as well as a decrease in inhibitory neurotransmission, resulting in a state of continuous hyperexcitability [436]. 

Until recently, epilepsy treatment was primarily focused in stopping seizures, disregarding the underlying mechanisms behind the disease. Nonetheless, this paradigm is changing, with research converging into efforts on finding new anticonvulsants, with both neuroprotective and antiepileptic properties. According to recent data, the ECS and its constituents may represent such therapeutic targets [437,438]. In fact, epilepsy often induces alterations in the ECS, particularly at the level of CB1R expression and production of eCBs [437]. Indeed, current evidence in mice suggests that CB1R expression is upregulated at GABAergic synapses and downregulated at glutamatergic synapses in epilepsy, although no consensus has been reached [439,440]. Moreover, epilepsy in humans affects the production of endogenous eCBs by interfering with the levels of cannabinoid enzymes like DAGL and MAGL, which is suggestive of a pivotal role of cannabinoid tone in this disease [441,442]. Emerging clinical evidence, mostly coming from epidemiological data and case reports, depicts the overall positive effects of cannabinoid administration using a high ratio of CBD:THC in the management of resistant epilepsy [443,444,445]. Furthermore, cannabinoids and their endogenous counterparts have been associated with epilepsy treatment, with several studies using various cannabinoid-based drugs in animal models of epilepsy [446,447,448,449,450,451]. For example, treatment with WIN 55,212-2 (a CB1R/CB2R agonist) was shown to prevent chronic epileptic hippocampal damage in rats by attenuating the severity and frequency of spontaneous recurrent seizures [452]. In particular, studies using CB1R ligands have shown that the activation of this receptor can delay the progression of seizure severity as well as the frequency of spontaneous epileptiform activity [446,447,450]. Moreover, studies using conditional CB1R KO models have demonstrated that eCB signaling plays an important role in the termination of epileptic activity, depending on the neuronal subpopulation, whilst having no impact in the initiation of hyperexcitability [451]. Lastly, it has also been shown that the MAGL inhibition leads to a delay in the development of generalized seizures in the kindling model of temporal lobe epilepsy [449]. Apart from their intrinsic anticonvulsant properties, cannabinoids have been shown to potentiate other anti-epileptic drugs [453,454,455].

Importantly, epileptic seizures were shown to promote aberrant AHN in the granular layer, characterized by a transient increase in the proliferation of neural progenitors, GCL dispersion, persistence of hilar basal dendrites and ectopic placing of adult-born GCs [430,435]. Evidence shows that prolonged seizures induce an increase in cell proliferation in the SGZ (up to 5–10 fold), lasting for several weeks [435,456,457,458]. However, approximately three to four weeks after the persistent seizure period, proliferation returns to baseline levels or even decreases to substantially lower rates when compared to control animals [435,457,459]. The same has been observed in humans, where it was described that severe seizures during early childhood prompt a decrease in AHN, negatively affecting normal brain development and further progression of epileptogenesis [460]. Whether the mechanism in patients is similar to those found in animal models, i.e., a transient increase in the proliferation of neural progenitors followed by a reduction of neurogenesis, is not known, instigating further studies to address this matter [435,460,461].

The alterations in neurogenesis go beyond cell proliferation, also affecting maturation and migration of adult-born neurons. After status epilepticus, which consists of a single epileptic seizure lasting more than five minutes or two or more seizures within a five-minute period without recovery of consciousness, newborn GCs migrate towards the dentate hilus or the molecular layer instead of integrating into the GCL, both in rodent models and patients with epilepsy [457,459,462,463,464]. Moreover, the correct maturation of the GCs upon epileptic stimuli does not occur, being observed an accumulation of hilar basal dendrites, which are normally a feature of immature cells. This abnormal maturation may be one of the mechanisms underlying the hyperexcitability of adult-born GCs and the circuits where they integrate [465,466,467,468]. Importantly, cannabinoids, when combined with antiepileptic drugs, can increase neurogenesis in the pilocarpine mouse model of epilepsy [469,470]. In fact, co-administration of ACEA, a selective CB1R agonist, with sodium valproate, a classic antiepileptic drug, was shown to significantly increase the number of proliferating cells in the same model [469,470]. However, further studies are needed to ascertain whether this increase in neurogenesis is not aberrant and can contradict the disease symptoms. Nonetheless, these results show promise by suggesting that NSC modulation by cannabinoids can be a potential target in this disorder.

As aforementioned, epilepsy treatment is an evolving and emerging topic with the search for new drugs and therapeutic targets ever increasing [471]. One key aspect that can be targeted is the seizure-induced neurogenesis, which can also help ameliorate the disease comorbidities [472]. Indeed, targeting aberrant AHN may reduce recurrent seizures and restore cognitive deficits, namely memory impairment [473,474,475]. Since it is known that the ECS can, on one hand, regulate adult neurogenesis and, on the other hand, have an impact in epileptic treatment, further studies are required to investigate the putative mechanisms by which cannabinoids have an impact in the treatment of epilepsy. Moreover, understanding how cannabinoid-induced modulation of NSCs may have neuroplastic effects and whether this can be used as an anti-epileptic treatment is highly relevant.

#### 5.2.5. Anxiety/Depression

Anxiety and depression are neuropsychiatric conditions with high prevalence worldwide, its symptoms range from irritability, anhedonia, difficulties in concentrating as well as disturbances in appetite, sleep, decreased productivity and increased suicide risk [476].

Alterations at the level of NSCs, especially in the hippocampus, are well known correlates of both anxiety and depressive disorders [477]. The ECS is a known modulatory key player in NSC regulation, drugs targeting this system induce mood alterations [478,479]. On the other hand, AHN has been shown to be required for the effects of antidepressants, suggesting that facilitation of neurogenesis can be beneficial for chronic antidepressant treatment [480]. In line with this, several findings suggest the involvement of cannabinoids in these neurogenesis-promoted long-lasting antidepressant effects [481]. Similar to the actions of conventional antidepressants, cannabinoid modulation was shown to promote antidepressant and anxiolytic effects [479,482]. Therefore, in recent years there has been a marked increase in the interest of using the ECS as a potential therapeutic target in these disorders [213,481,483,484].

Interestingly, changes in eCB levels have also been reported in affective disorders. The circulating levels of these molecules have been found to be diminished in individuals diagnosed with depressive and anxiety disorders [485,486]. Likewise, in animal models of depressive-like behavior there is a significant overall decrease in brain AEA levels, suggesting an impairment of eCB tone [487,488,489]. In line with this, polymorphisms in the gene encoding for FAAH (*FAAH*), have been linked to an increased risk of depressive and anxiety disorders [490,491]. Moreover, in animal models of depressive-like behavior, restraint stress induces a significant increase of FAAH expression in numerous brain regions associated with affective functioning [492,493,494]. Data regarding 2-AG is less clear: while it was found to be diminished in the some regions, as a consequence of chronic mild stress (CMS), it has been observed to be increased in several key regions of the limbic system such as the amygdala, in response to the same stress exposure [492,493,494]. In accordance with this last finding, MAGL expression has been found to decrease over time, in response to persistent stress [494]. These results have led some researchers to propose that 2-AG production may be stimulated in situations of persistent stress, as a buffer mechanism against possible stress-induced neuronal dysregulation [494,495].

Acute and intermittent administration of CB1R agonists is known to biphasically modulate anxiety, in both humans and rodents. Low doses are known to be anxiolytic, and higher doses anxiogenic [496,497]. At the behavior level, acute administration of CB1R agonists such as Δ^9^-THC, CBC, ACEA, HU-210, CP 55,940 and WIN 55,212-2, have been mostly related to improvements in the performance of animals in several behavioral tasks, as well as the overall phenotype of animal models of depressive-like behavior [498,499,500,501,502,503,504]. In contrast, chronic exposure to CB1R agonists have been shown to induce marked alterations that are age- and gender-dependent [505,506]. Specifically, epidemiological data suggests that while adult chronic use may be a risk factor for anxiety and depressive disorders, this deleterious effect is not as pronounced as in adolescents [507,508,509,510]. Indeed, chronic adolescent users have been consistently found to have a higher risk of being diagnosed with anxiety and/or depressive disorders, and that this risk is bigger in females [511,512,513]. Curiously, in animal models, the opposite is suggested, since in adolescent animals no persisting alterations at the level of anxiety have been found but depressive-like behavior has been found to be markedly increased [505,506,514]. Importantly, this lasting impairment in depressive-like behavior seems to be largely restricted to female animals, and is accompanied by impairments in hippocampal neurogenesis, which is reversed by treatment with the FAAH inhibitor URB597 [506,515,516,517]. In contrast, adult animals chronically exposed to potent full agonists of cannabinoid receptors show persisting anxiolytic and antidepressant effects, along with enhanced AHN, underscoring the importance of age in the long-term effects of cannabinoids [518,519,520,521].

The effects of CB1R antagonism/inverse agonism are much more consistent. Despite some animal behavioral evidence of anxiolytic- and antidepressant-like effects, rimonabant (SR141716) and AM251 have been well described as promoters of anxiety and depressive-like symptoms in both rodents and humans when chronically administered [494,522,523,524,525,526,527]. Indeed, rimonabant, initially commercialized for the treatment of obesity, was recalled after being related to increases in depressive/anxious symptoms [528]. Additionally, in rodents, this compound has been demonstrated not only to promote depressive-like behavior, but also to reduce cell proliferation and survival in the hippocampus [526]. 

There is evidence suggesting that single nucleotide polymorphisms in the CB1R coding gene (*CNR1*) are related to an increased risk of stress-precipitated depressive episodes, as well as resistance to antidepressant drugs [529,530]. In accordance, these polymorphisms are more prevalent among individuals diagnosed with mood disorders [491]. In line with this, CMS induces a decrease in CB1R expression in the hippocampus, hypothalamus and striatum [489,531]. Furthermore, CB1R KO rodents present a characteristic anxious/depressive-like behavioral profile, accompanied by a 50% decrease in hippocampal NSC proliferation [213,525,532].

Little research has been published so far on the effects of CB2R agonists on anxiety- and depressive-like behaviors, with a few contradictory reports. Some authors report CB2R agonists to have anxiolytic effects (JWH-015 and BCP), others report anxiogenic effects (JWH-133), contrasting with others that report no effects (GW405833) [533,534,535,536,537,538]. Moreover, CB2R agonists like BCP, JWH-133 and oleamide, may have antidepressant-like effects, despite some reports finding no changes with the treatment (JWH-015) [533,534,539]. Curiously, given the effects of CB2R activation described above, there are reports showing CB2R antagonist AM630 as having anxiolytic and antidepressant-like effects [538,539,540]. 

Likewise, not much is known about the involvement of CB2Rs in the pathophysiology of depressive and anxiety disorders. In humans, a study found an association between a polymorphism in the CB2R coding gene (*CNR2*) and a number of psychiatric and immune disorders, which are often comorbidities [541]. Moreover, animals carrying this polymorphism are less sensitive to the CB2R-mediated effects of WIN 55,212-2 and 2-AG, suggesting a possible link between impaired CB2R functioning and altered behavioral phenotypes [542]. In fact, CB2R KO animals present increased anxiety- and depressive-like behaviors [543,544]. Similarly, animals overexpressing CB2Rs not only show reduced anxious- and depressive-like behavioral phenotypes, but also are actually resistant to the deleterious effects of CMS exposure [540]. Furthermore, CMS has been shown to lead to decreased CB2R mRNA levels in whole brain samples, as well as in hippocampal homogenates [537,540,541]. 

Given the psychoactive side-effects of CB1R agonists, one increasingly popular approach has been the modulation of the enzymes responsible for the degradation of eCBs, such as FAAH and MAGL [545]. This approach has, indeed, shown some significant promise, with inhibition of FAAH and to a lesser extent MAGL, leading to anxiolytic- and antidepressant-like effects in rodents [546,547,548,549,550,551,552,553]. Importantly, in the CMS model of depression, the MAGL inhibitor JZL184 was shown to rescue AHN [551].

CBD has also been extensively shown to have potent anxiolytic and antidepressant effects in both humans and rodents, being currently investigated as a possible new avenue for the treatment of these disorders (reviewed in [554,555,556]). As such, acute and chronic CBD administration have been shown to induce anxiolytic- and antidepressant-like changes in animal behavioral tests, with low doses resulting in anxiolytic effects, while higher doses produce no effect on anxiety [499,557,558,559,560,561,562,563]. In addition, CBD-induced anxiolytic effects were shown to be dependent on AHN [220].

In summary, there is significant evidence supporting the assertion that the ECS positively modulates AHN, possibly having a critical role on the regulation of affective states. Moreover, the importance of this system in these processes is further underlined by the effects that pharmacological modulation has on the ECS on indexes of mood, in both animals and humans.

## 6. Perspectives and Concluding Remarks

In recent years there has been a growing increase in the number of reports highlighting the effects of cannabinoids on NSC regulation [180,210,231]. With the existence of several plant-derived and synthetic cannabinoids with little or no psychoactive properties, there is an enormous uncovered potential for cannabinoids as treatment for several neurological disorders, especially when combined with stem cell therapy [444,564].

**Figure 4:**
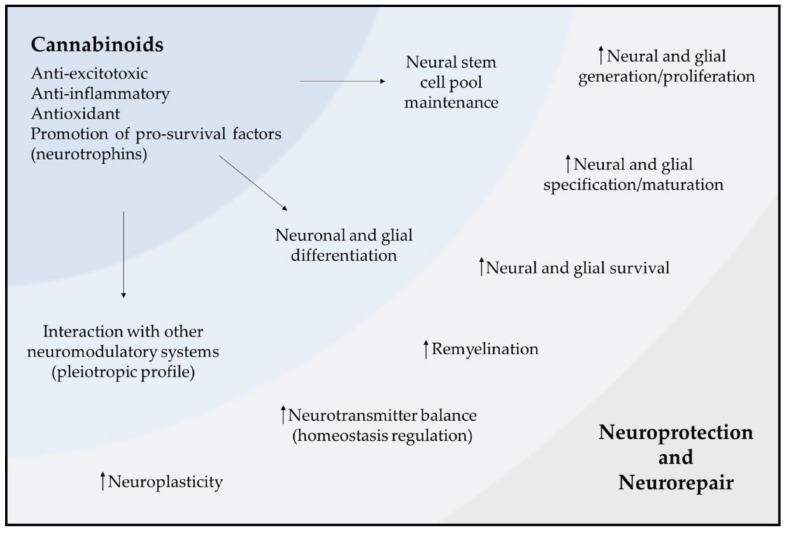
Cannabinoid actions. Cannabinoids exert a pivotal role in controlling neural stem cell dynamics by acting on vital intrinsic/extrinsic signaling pathways, therefore regulating distinct steps of neural stem cells proliferation, differentiation and maturation, namely by maintaining the pool of neural stem cells, promoting neuronal and glial differentiation or by interacting with other neuromodulatory systems. The anti-excitotoxic, anti-inflammatory and antioxidant effects of cannabinoids modulate neuroplasticity, homeostasis, remyelination, survival, maturation and proliferation of both glial and neuronal cells. When taking in account all of these factors, cannabinoids are shown to be extremely versatile molecules with neuroprotective and repair capabilities within the central nervous system.

In the present review, we put forth a detailed view of how the ECS acts as a potent regulator of NSC biology by presenting a large body of evidence supporting the effects of cannabinoids on NSCs and their potential to modulate several neurogenic features in the context of pathophysiology (Figure 4 and Supplementary Table S1). In fact, endogenously-produced, plant-derived or synthetic cannabinoids regulatory actions upon NSC pool maintenance and neuronal/glial proliferation and differentiation constitute a potential mechanism for the treatment of brain disorders since (1) neuronal damage and neuroinflammation are often associated with alterations in the ECS and (2) hypofunction or dysregulation of the ECS seems to be correlated with some symptoms and neurobiological hallmarks of several brain disorders [56,214,565,566]. In particular, stimulation of NSC proliferation and survival in both the embryonic and adult brain has been shown to be beneficial, for example, in AD, PD, MS, epilepsy and anxiety/depression [331,361,420,435,481,567]. 

Cannabinoid-based treatment is a very promising avenue of therapy due to the wide distribution of the ECS throughout the CNS and its complex interactions with other neuromodulatory systems, although these represent challenging factors *per se* when it comes to designing neural repair approaches. Moreover, the multiplicity of cannabinoid actions on NSCs, particularly in disease context, opens avenues of research to uncover the exact mechanisms behind cannabinoid effects, therefore, providing knowledge that should be invested in developing refined targeting strategies to alleviate/arrest disease burden. Based on the evidence presented in this review regarding neurogenesis and the relevance of the ECS in maintaining CNS homeostasis, cannabinoid-based medicines may represent promising therapeutic tools to tackle several brain disorders. There is still, however, a huge lack of pre-clinical, clinical reports and case studies attesting the efficacy of cannabinoids in pathological contexts. Pre-clinical data still fails to deliver the precise mechanisms by which cannabinoids and ECS modulation impact disease neurobiology and phenotypic progression. On the other hand, the few existing clinical evidence comes either from patients without a cannabis-consuming background or from those who started using cannabis as a last resort when all other conventional therapies had failed [568]. Additionally, at the conceptual level, several questions remain to be addressed, including the relevance of cannabinoid-mediated actions through “non-canonical” or orphan receptors (that is, non-CB1 and non-CB2 receptors like GPR55, GPR18, PPARγ receptors or TRPV channels) as well as the precise signaling mechanisms by which cannabinoids control neurogenesis and mediate disease states. Given the existence of a plethora of pre-clinically tested, synthetic, botanical and multi-target cannabinoid-based drugs, the identification of a cannabinoid gold standard to be used as a clean and reliable clinical therapeutic option is still missing [259,569]. Concomitantly, the wide array of cannabinoid actions makes the process of choosing the best cannabinoid-based compound with safer clinical effects difficult. Moreover, it is important to weigh in to which extent cannabinoids can be used as therapeutic weapons by taking into consideration the benefit-to-risk ratio in a clinical setting and the narrow therapeutic window exhibited in some disease phenotypes.

With more and more countries legalizing the use of cannabinoids for medicinal purposes, surely there will be a push from drug developers and pharma industry to do more pre-clinical and clinical studies in the future. Overall, the abovementioned unexplored questions reveal that we are still scraping the tip of the iceberg when it comes to the use of cannabinoids as medicinal agents and further research on the precise roles of cannabinoid signaling in NSC biology is needed. Combining both cannabinoid and NSC therapy may yield translational power with potential to be impactful in clinics and society. Certainly, new knowledge in this field over the upcoming years will bring forth innovative ideas that could place cannabinoids as one of the leading pharmacotherapeutic options to treat brain pathology.

## Supplementary Materials

The following are available online. Table S1: Modulation of the Endocannabinoid System in pathophysiological conditions title.

## Abbreviations

(*N*-acyl-phosphatidylethanolamine)-specific phospholipase D	NAPE-PLD
2-arachidonoglycerol	2-AG
6-hydroxydopamine	6-OHDA
Acetylcholinesterase	AChE
Adult hippocampal neurogenesis	AHN
Alpha-synuclein	αSyn
Alzheimer’s disease	AD
Amyloid precursor protein/presenilin 1	APP/PS1
Amyloid-β	Aβ
Anandamide	AEA
Brain-derived neurotrophic factor	BDNF
Cajal-Retzius cell	CRC
Calcium-dependent trans-acylase	NAT
Cannabichromene	CBC
Cannabidiol	CBD
Cannabidivarin	CBDV
Cannabigerol	CBG
Cannabinoid receptor type 1	CB1R
Cannabinoid receptor type 2	CB2R
Cannabinol	CBN
Cannabivarin	CBV
CB1R interacting protein 1a	CRIP1a
Central nervous system	CNS
Chronic mild stress	CMS
Cornu Ammonis 3	CA3
Dentate Gyrus	DG
Depolarization-induced suppression of excitation	DSE
Depolarization-induced suppression of inhibition	DSI
Diacylglycerol lipase	DAGL
Dopaminergic	DA
Doublecortin	DCX
Endocannabinoid membrane transporter	EMT
Endocannabinoid system	ECS
Endocannabinoids	eCBs
Epidermal growth factor	EGF
Experimental autoimmune encephalomyelitis	EAE
Fatty acid amide hydrolase	FAAH
Fibroblast growth factor	FGF
G protein-coupled receptors	GPCR
Glial fibrillary acidic protein	GFAP
Glycogen synthase kinase 3β	GSK-3β
Granule cell layer	GCL
Granule cell	GC
Human immunodeficiency virus infection and acquired immune deficiency syndrome	HIV/AIDS
Interferon-γ	IFN-γ
Interleukin 1	IL-1
Intermediate progenitor cells	IPC
Long-term depression	LTD
Molecular layer	ML
Monoacylglycerol lipase	MAGL
Multiple sclerosis	MS
N-arachidonoyl-phosphatidyl ethanolamine	NArPE
Nerve growth factor	NGF
Neural stem cells	NCS
Neuroepithelial progenitor cells	NEC
Neurotrophin-3	NT-3
Olfactory bulb	OB
Oligodendrocyte progenitor cells	OPC
Parkinson’s disease	PD
Peripheral nervous system	PNS
Peroxisome proliferator-activated receptor	PPAR
Phosphatidylinositol 3-kinase	PI3K
Phospholipase A1	PLA1
Phospholipase C	PLC
Polysialic acid neural cell adhesion molecule	PSA-NCAM
Preplate	PP
Protein kinase C	PKC
Radial glial cell	RGC
Reactive oxygen species	ROS
Rostral migratory stream	RMS
Short-term depression	STD
Sonic hedgehog	shh
Striatum	STR
Subgranular Zone	SGZ
Substantia nigra pars compacta	SN
Subventricular Zone	SVZ
Theiler murine encephalomyelitis virus-induced demyelinating disease	TMEV-IDD
Transient receptor potential vanilloid receptor type 1	TRPV1R
Tumor necrosis factor α	TNF-α
Ventricular zone	VZ
β-caryophyllene	BCP
Δ^8^-tetrahydrocannabinol	Δ^8^-THC
Δ^9^-tetrahydrocannabinol	Δ^9^-THC
Δ^9^-tetrahydrocannabivarin	THCV
