# Supplementary material for: Cannabinoid Actions on Neural Stem Cells: Implications for Pathophysiology
Source: Molecules. 2019 Apr 5;24(7):1350. doi: 10.3390/molecules24071350 (PMC6480122; doi:10.3390/molecules24071350)
Supplement: Supplementary file 1 [file molecules-24-01350-s001.pdf]

Supplementary Table 1 – Modulation of the Endocannabinoid System in pathophysiological conditions.

	Models	Cannabinoid Modulation	Effects
Alzheimer's Disease	Microglial cell cultures (mice) + Aβ1-42	JWH-015	↓ Production of proinflammatory cytokines; ↑ Aβ phagocytosis [243]
	Microglia cell cultures (rat) + Aβ1-40	WIN 55,212-2; JWH-133; HU-210	↓ Microglial activation [275]
	Microglia-neuron co-cultures (rat) + Aβ1-40	WIN 55,212-2; JWH-133	↓ Microglial induced neurotoxicity by ↑ neuronal survival [275]
	Hippocampal neuron cultures (rat) + Aβ25-35/Aβ1-42	2-AG; URB602, JZL184	↓ Neurodegeneration and apoptosis [289]
	Cortical neuron cultures (rat) + Aβ1-42	AEA	↑ Notch-1 signalling [316]
	PC12 cells (rat) + Aβ1-40		↓ Neuronal cell loss [290]
	PC12 cells (rat) + Aβ1-42	CBD	↑ Cell survival; ↓ ROS production, lipid peroxidation [292]
	SHSY5Y cell cultures (human) + Aβ1-42		↓ Aβ neurotoxicity [296]
	SHSY5Y(APP+) cell cultures (human)		↓ Aβ production; ↑ Cell survival [293]
	HEK(APP+) cell cultures (human); mixed glia-neuron cultures (mice) transfected with APPswe mutation	PPARγ activation	↓ APP expression; ↑ Aβ clearance [294,295]
	Icv Aβ1-42 injection (mice); Intracortical Aβ1-42 injection (rats)	VDM-11	↓ Hippocampal neuronal damage (rats); memory impairment (mice) [276]
	Icv Aβ1-40 injection (mice); Icv Aβ25-35 injection (rats)	CBD (mice); WIN 55,212-2 (rats)	↓ Microglial activation; spatial learning/memory impairment [244,275]
	Intrahippocampal Aβ1-42 injection (rats)	WIN 55,212-2	↓ Neuroinflammation; spatial learning/memory impairment [298]
	Intrahippocampal Aβ1-42 injection (rats/mice)	CBD	↓ Reactive gliosis; rescued neuronal integrity; repaired AHN [201,302]
	APPswe mice	JWH-133	↓ Microglial activation, COX-2, TNF-α mRNA, Aβ levels, memory deficits [245]
		WIN 55,212-2	↓ GSK3β activity and levels of Aβ [245]
	5xFAD mice	Δ^9-THC; JZL184	↓ Occurrence of neuritic plaques [307,308]
	APP/PS1 mice	MAGL genetic ablation; JZL184	↓ Reactive gliosis, pro-inflammatory cytokines, density of Aβ plaques; spatial learning/memory impairment [297]
		ACEA	↓ GSK3β, tau phosphorylation, astrogliosis, cognitive impairment; no effect on Aβ production, aggregation or clearance [299]
		MDA7	↓ Microgliosis; ↑ Aβ clearance, hippocampal Sox2 expression, synaptic plasticity, spatial learning/memory [300]
		JWH-133	↓ Microgliosis, pro-inflammatory cytokines, GSK3β activity, tau phosphorylation, cognitive impairment; no effect on Aβ production [301]
		Δ^9-THC; CBD	↑ Memory performance [303,304]
		Δ^9-THC+CBD	↓ Soluble Aβ1-42, reactive gliosis, learning impairments (young animals); ↓ Memory deficits with no effect on Aβ burden or reactive gliosis (aged animals) [303,304]
		CBD	↓ Memory deficits; no effect on Aβ burden [305,306]
	Post-mortem human samples	JWH-015	↑ Aβ phagocytosis [291]

Human trials	Dronabinol	↓ Aggressive behaviour, ↑ body weight [268]; ↓ Motor agitation [269]; ↓ Nocturnal motor activity [270,271]

Supplementary Table 1 – Continuation

Parkinson's Disease	Human cord blood iPSCs	Δ^9-THC, AEA	Concentration-dependent actions of cannabinoids on neuronal function [190]
	SVZ neurospheres (mice)	R-m-AEA	No increase in TH-positive cells [198]
	Cerebellar granule cell cultures (mice) + 6-OHDA / unilateral injection of 6-OHDA	Δ^9-THC; CBD; HU-210	↑ Neuroprotection [341]
	Rotenone-induced rat model	BCP; AM-630	↑ Neuroprotection and antioxidant properties [342]
	Human trials	Cannabis	↓ Bradykinesia, muscle rigidity and tremors [329]
Multiple Sclerosis	Splenocytes cell cultures (mice) / EAE mice	CBD	↓ EAE severity;↓ Pro-inflammatory cytokines; ↑ Anti-inflammatory cytokine release [384]
	EAE mice	CBD	↓ Inflammatory immune cells; ↓ Demyelination [406]
		Δ^9-THC; CBD	↓ IL-17 and IL-6 secretion; ↑ IL-10 secretion [385]
		Gp1a	↓ Clinical scores and $CD4^+$ T cells in the CNS [388]
		WIN 55,212–2; SR141716; SR144528	↓ Leukocyte/endothelial cell interaction and leukocyte trafficking into the CNS [387]
		Δ^9-THC; CB1R KO in neurons or T cells and CB2RKO mice	Amount of CB1R and CB2R activation determined EAE severity; CB2R as a regulator of EAE clinical disease in the steady state [379]
		CB1R KO	↑ Neurodegeneration and axonal loss; ↓ Anti-inflammatory responses [377]
	TMEV-IDD mouse model	UCM707	↑ Motor function; ↓ Microglia/macrophage activation, pro-inflammatory cytokine release [383]
	Human trials	Cannabis	↓ Spasticity, pain relief [389,399]
Epilepsy	Pilocarpine mouse model	Δ^9-THC, WIN 55,212-2, SR141716	Delayed progression of seizure severity and frequency of spontaneous epileptiform activity [429]
		ACEA + anti-epileptic drugs	↑ Hippocampal neurogenesis [452,453]
	Pilocarpine rat model	WIN 55,212-2	Attenuated severity, duration and frequency of spontaneous recurrent seizures [435]
	Penicillin-induced epileptiform activity rat model	AM-251, ACEA	Modulation of frequency of epileptiform activity [430]
	PTZ-induced clonic seizure mice	WIN 55,212-2 + anti-epileptic drugs	↑ Anticonvulsant activity [436, 437, 438]
	Maximal electroshock-induced seizures mice	ACEA, PMSF + anti-epileptic drugs	
	Kindling mouse model of temporal lobe epilepsy	JZL184	Delayed development of generalized seizures, without acute anticonvulsive effects [432]
		WIN 55,212-2	Delayed progression of seizure severity [433]
		URB597	↓ Neurogenesis [433]
		CB1R KO; SR141716	eCBs signalling affected the termination of epileptic activity [434]

Supplementary Table 1 – Continuation

Anxiety/Depression	Wild-type mice	CP 55,940	Anxiolytic- (low dose) or anxiogenic-like effects (high dose) [479]
		CP 55,940; WIN 55,212-2; AM404; URB597	Anxiolytic-like effects [507, 508, 516, 521, 536]
		SR141716	
		JWH-015	
		AM630	
		URB597; JZL184	
		Δ^9-THC; SR141716; AM251	Anxiogenic-like effects [507, 521]
		AM630; JWH-133	
		CBD	Antidepressant-like effects [481, 482, 484, 522, 523, 531, 543–545]
		Oleamide; JWH-133; AM630	
		Δ^9-THC; CBC; CBD; ACEA; SR141716	
		PF3845	
		BCP	Antidepressant and anxiolytic-like effects [517, 529]
		JZL184; KML29	
		URB597; FAAH KO	Anxiolytic-like effects [505, 530]
		SR141716; CB1R KO	
		CB1R KO	Anxiogenic-like effects [508]
		CB2R KO	Anxiogenic- and depressive-like behaviours [526,527]
		CB2R overexpression	Antidepressant- and anxiolytic-like effects [523]
	Wild-type rats	AM404; HU-210; Oleamide; WIN 55,212-2	Antidepressant-like behaviour [483,501,502]
		WIN 55,212-2	Anxious-like behaviour and spatial learning/memory deficits; ↓ Dorsal hippocampal neurogenesis [498]
		URB597	Rescued Δ^9-THC-induced depressive-like behaviour and deficits in AHN [499]
		HU-210	↑ Response to stress ↓ hippocampal neurogenesis [500]; Antidepressant- and anxiolytic-like effects, ↑AHN [503]
		SR141716	Anxiogenic-like effects [506]; Induced depressive-like behaviours, ↓ AHN, cell survival [509]
		CBD	Antidepressant-like effects; anxiolytic-like effects [541]

Supplementary Table 1 – Continuation

Anxiety/Depression	Wild-type mice; CMS mouse model of depression	CB1R KO	↑ Aggressive, anxiogenic- and depressive-like behaviours [515]
	Wild-type rats; CMS mouse model of depression	SR141716	Antidepressant-like effects [505]
	CMS mouse model of depression	JZL184	Antidepressant- and anxiolytic-like effects[534,535]; rescue of AHN [534]
		CBD	Anxiolytic-like effects; Promoted AHN [203]
	CMS rat model of depression	WIN 55,212-2	Prevented emotional learning deficits and LTP impairment [487]
		URB597	Antidepressant-like effects [532]
	Olfactory bulbectomy mouse model of depression	CBD	Antidepressant and anxiolytic-like effects [540]
	Olfactory bulbectomy rat model of depression	Δ^9-THC; SR141716	Antidepressant-like behaviour [485]
	Restraint stress mouse model of depression	CP 55,940; URB597	Antidepressant-like behaviour [486]
	FSL and LH rat models of depression	CBD	Antidepressant-like effects [545]
	Human trials	Cannabis (chronic use)	↑ Risk of suffering from anxiety and/or depressive disorders [490,492,494–496]
		SR141716	↑ Depressive symptoms [510,511]
		CBD	Induced anxiolytic effects [546]

Abbreviations: (2-Methyl-1-propyl-1*H*-indol-3-yl)-1-naphthalenylmethanone (**JWH-015**); [2,3-Dihydro-5-methyl-3-(4-morpholinylmethyl)pyrrolo[1,2,3-de]-1,4-benzoxazin-6-yl]-1-naphthalenylmethanone mesylate (**WIN55,212–2**); (6a*R*,10a*R*)-3-(1,1-Dimethylbutyl)-6a,7,10,10a-tetrahydro-6,6,9-trimethyl-6*H*-dibenzo[*b,d*]pyran (**JWH-133**); (6a*R*)-*trans*-3-(1,1-Dimethylheptyl)-6a,7,10,10a-tetrahydro-1-hydroxy-6,6-dimethyl-6*H*-dibenzo[*b,d*]pyran-9-methanol (**HU-210**); 2-Arachidonoylglycerol **(2-AG**); *N*-[1,1'-Biphenyl]-3-yl-carbamic acid cyclohexyl ester (**URB602**); 4-[*Bis*(1,3-benzodioxol-5-yl)hydroxymethyl]-1-piperidinecarboxylic acid 4-nitrophenyl ester (**JZL184**); Anandamide (**AEA**); Cannabidiol (**CBD**); Peroxisome proliferator-activated receptor γ (**PPARγ**); (5*Z*,8*Z*,11*Z*,14*Z*)-*N*-(4-Hydroxy-2-methylphenyl)-5,8,11,14-eicosatetraenamide (**VDM-11**); Δ^9-tetrahydrocannabinol (**Δ^9-THC**); *N*-(2-Chloroethyl)-5*Z*,8*Z*,11*Z*,14*Z*-eicosatetraenamide (**ACEA**); 1-[(3-benzyl-3-methyl-2,3-dihydro-1-benzofuran-6-yl)carbonyl]piperidine (**MDA7**); (*R*)-(+)-Methanandamide (**R-m-AEA**); β-caryophyllene (**BCP**); 6-Iodo-2-methyl-1-[2-(4-morpholinyl)ethyl]-1*H*-indol-3-yl](4-methoxyphenyl)methanone (**AM-630**); *N*-(Piperidin-1-yl)-1-(2,4-dichlorophenyl)-1,4-dihydro-6-methylindeno[1,2-c]pyrazole-3-carboxamide (**Gp1a**); *N*-(Piperidin-1-yl)-5-(4-chlorophenyl)-1-(2,4-dichlorophenyl)-4-methyl-1*H*-pyrazole-3-carboxamide hydrochloride (**SR141716**); 5-(4-Chloro-3-methylphenyl)-1-[(4-methylphenyl)methyl]-*N*-[(1*S*,2*S*,4*R*)-1,3,3-trimethylbicyclo[2.2.1]hept-2-yl]-1*H*-pyrazole-3-carboxamide (**SR144528**); (5*Z*,8*Z*,11*Z*,14*Z*)-*N*-(3-Furanylmethyl)-5,8,11,14-eicosatetraenamide (**UCM707**); *N*-(Piperidin-1-yl)-5-(4-iodophenyl)-1-(2,4-dichlorophenyl)-4-methyl-1*H*-pyrazole-3-carboxamide (**AM-251**); Phenylmethylsulfonyl fluoride (**PMSF**); Cyclohexylcarbamic acid 3'-(Aminocarbonyl)-[1,1'-biphenyl]-3-yl ester (**URB597**); (-)-*cis*-3-[2-Hydroxy-4-(1,1-dimethylheptyl)phenyl]-*trans*-4-(3-hydroxypropyl)cyclohexanol (**CP55,940**); *N*-(4-Hydroxyphenyl)-5*Z*,8*Z*,11*Z*,14*Z*-eicosatetraenamide (**AM404**); Cannabichromene (**CBC**); Fatty Acid Amide Hydrolase piperidine urea inhibitor (**PF3845**); 4-[*Bis*(1,3-benzodioxol-5-yl)hydroxymethyl]-1-piperidinecarboxylic acid 2,2,2-trifluoro-1-(trifluoromethyl)ethyl ester (**KML29**)
